# Mechanism on the promotion of host growth and enhancement of salt tolerance by *Bacillaceae* isolated from the rhizosphere of *Reaumuria soongorica*

**DOI:** 10.3389/fmicb.2024.1408622

**Published:** 2024-05-31

**Authors:** Xinguang Bao, Peifang Chong, Cai He, Xueying Wang, Feng Zhang

**Affiliations:** ^1^College of Forest of Gansu Agriculture University, Lanzhou, China; ^2^Wuwei Academy of Forestry, Wuwei, China

**Keywords:** *Reaumuria soongorica*, plant growthpromoting rhizobacteria (PGPR), salt stress, plant hormones, metabolites

## Abstract

Salt stress is a major abiotic stress that affects the growth of *Reaumuria soongorica* and many psammophytes in the desert areas of Northwest China. However, various Plant Growth-Promoting Rhizobacteria (PGPR) have been known to play an important role in promoting plant growth and alleviating the damaging effects of salt stress. In this study, three PGPR strains belonging to *Bacillaceae* were isolated from the rhizosphere of *Reaumuria soongorica* by morphological and molecular identification. All isolated strains exhibited capabilities of producing IAA, solubilizing phosphate, and fixing nitrogen, and were able to tolerate high levels of NaCl stress, up to 8–12%. The results of the pot-based experiment showed that salt (400 mM NaCl) stress inhibited *Reaumuria soongorica* seedlings’ growth performance as well as biomass production, but after inoculation with strains P2, S37, and S40, the plant’s height significantly increased by 26.87, 17.59, and 13.36%, respectively (*p < 0.05*), and both aboveground and root fresh weight significantly increased by more than 2 times compared to NaCl treatment. Additionally, inoculation with P2, S37, and S40 strains increased the content of photosynthetic pigments, proline, and soluble protein in *Reaumuria soongorica* seedlings under NaCl stress, while reducing the content of malondialdehyde and soluble sugars. Metabolomic analysis showed that strain S40 induces *Reaumuria soongorica* seedling leaves metabolome reprogramming to regulate cell metabolism, including plant hormone signal transduction and phenylalanine, tyrosine, and tryptophan biosynthesis pathways. Under NaCl stress, inoculation with strain S40 upregulated differential metabolites in plant hormone signal transduction pathways including plant hormones such as auxins (IAA), cytokinins, and jasmonic acid. The results indicate that inoculation with *Bacillaceae* can promote the growth of *Reaumuria soongorica* seedlings under NaCl stress and enhance salt tolerance by increasing the content of photosynthetic pigments, accumulating osmoregulatory substances, regulating plant hormone levels This study contributes to the enrichment of PGPR strains capable of promoting the growth of desert plants and has significant implications for the psammophytes growth and development in desert regions, as well as the effective utilization and transformation of saline-alkali lands.

## 1 Introduction

Data indicate that the area of saline soils worldwide exceeds 833 million hectares, accounting for 8.7% of the Earth’s surface area from the Global Saline Soils Map published by the Food and Agriculture Organization of the United Nations in 2021. China’s saline soil totals 36.9 million hectares, comprising 4.4% of the global saline soil (Yang et al., 2022). Saline soils are mainly distributed in arid and semi-arid regions and have become a global issue affecting vegetation restoration and agricultural productivity. Under salt stress, the massive accumulation of Na^+^ and Cl^−^ within plant cells leads to ionic imbalance, the production of reactive oxygen species, oxidative damage, and disruption of plant metabolic functions, inhibiting plant growth (Kibria et al., 2017; Sunita et al., 2020). Various physical, chemical, hydraulic engineering, and biological methods are employed in agriculture to ameliorate saline soils. At present, microbial improvement of saline-alkali soils is recognized as one of the most environmentally friendly and sustainable measures and has become a hot research topic. Practical evidence has demonstrated that some beneficial microbes can promote plant growth and help plants resist biotic and abiotic stresses. Based on their effects on plant growth, nutrient cycling, and soil structure, beneficial microbes can be categorized into plant growth-promoting rhizobacteria (PGPR), nitrogen-fixing bacteria, arbuscular mycorrhizal fungi, and ectomycorrhizal fungi (Coban et al., 2022).

PGPR are soil bacteria that reside in the plant root vicinity and promote plant growth, either directly or indirectly, through their vital activities, helping plants cope with various biotic and abiotic stresses (Aguirre-Monroy et al., 2019; Gupta et al., 2019). Studies have found that PGPRs mediate the increase in soil nutrient availability and produce plant growth hormones to directly promote plant growth; for instance, nitrogen-fixing bacteria convert atmospheric nitrogen into plant-usable forms of NO3^−^ or NH4^+^ (Mukherjee and Sen, 2015), phosphate-solubilizing bacteria enhance soil phosphorus availability (Amri et al., 2023), siderophore-producing strains increase soil bioavailability of iron (Saha et al., 2016), and PGPR can promote plant growth by producing plant growth hormones, such as exogenous IAA, to stimulate root development and the formation of lateral and adventitious roots (Li et al., 2022). On the other hand, PGPR can indirectly support plant growth by suppressing the deleterious effects of biotic and abiotic stresses. For example, inoculation with *Azospirillum* and *Azotobacter* species significantly promoted growth and enhanced salt tolerance of cherry tomato under different salinity levels (El-Beltagi et al., 2022), and the addition of *Enterobacter cancerogenus* JY65 promoted the growth of rice under NaCl stress with significantly increased plant biomass, plant height, and root length (Peng et al., 2023). There is also evidence that plant secondary metabolites are involved (Sunita et al., 2020; Pang et al., 2021; Koza et al., 2022; Kumar et al., 2023). The *Bacillus subtilis* strain WM13-24 isolated from the rhizosphere of *Haloxylon ammodendron* promotes plant growth by producing volatile organic compounds that stimulate lateral root and root hair development (He et al., 2023). Flavonoids, as active molecules mediating communication between PGPR and plants, involve in PGPR-mediated plant tolerance to abiotic stresses, especially salinity stress (Wang et al., 2022). Thus, PGPR plays a key role in plant growth and development as well as in addressing various environmental stresses. Therefore, rhizosphere microbes certainly deserve as “second genome” of plants (Maeda and Dudareva, 2012).

However, competition exists between introduced microbes and indigenous microbial communities, and PGPR can only function effectively if they adapt to local soil environments (Al-Turki and Abdullah, 2021). Thus, many researchers have begun to isolate and screen new PGPR from special environments such as saline-alkali lands and arid deserts. Halotolerant PGPR strains isolated from salt mine soils induce salinity tolerance in wheat by enhancing the expression of SOS genes (Haroon et al., 2022). Similarly, halotolerant PGPR strains isolated from coastal saline soil improve nitrogen fixation and alleviate salt stress in rice plants (Khumairah et al., 2022). The *Enterobacter cancerogenus* JY65 isolated from extremely desert saline-lkali soil promotes the growth of rice under salt stress by producing IAA (Peng et al., 2023). Synthetic bacterial community derived from the root of the desert plant *Indigofera argenteaa* confer salt stress resilience to tomato (Schmitz et al., 2022). It is evident that PGPRs play a crucial role in the growth and stress resistance of crops, but research is primarily focused on crops, with few developments and research of PGPR for plants native to the northwest deserts. Through long-term monitoring, it has been found that *Reaumuria soongorica*, a small shrub of the *Tamaricaceae* family and a halophyte widely distributed in desert areas, is one of the typical dominant shrub species in the arid and semi-arid deserts of Northwest China (Ma et al., 2005; Bai et al., 2009). Therefore, this study isolated and screened PGPR strains with growth-promoting traits from the rhizosphere of the desert plant *Reaumuria soongorica,* and evaluated their growth-promoting effects and stress resistance on the host through pot experiments from physiological and metabolic perspectives. Elucidation of the mechanisms through which PGPRs promote plant growth and enhance salt tolerance holds immense importance for desert plant growth, as well as for bioremediation, advancement, and utilization of saline-alkali soil.

## 2 Materials and methods

### 2.1 Isolation and screening of PGPR strains

The rhizosphere soil of *Reaumuria soongorica* used in this experiment was collected in April 2023 from the Liangucheng National Nature Reserve in Minqin, Gansu. High-throughput cultivation and identification methods were employed to isolate and screen IAA-producing PGPR (Zhang et al., 2021). A sample of 10 g of soil was dissolved in 90 mL of sterile physiological saline and incubated on a shaker at 30°C, 180 r/min for 30 min. Gradient dilutions were prepared sequentially to produce 10^−3^, 10^−4^, and 10^−5^ dilutions. Various dilutions were inoculated into 96-well Lauria Bertani (LB) liquid culture plates, and after incubation, 30–40% of the wells exhibited visible bacterial growth, which indicated that each bacterial culture originated from a single cell, thus ensuring a high proportion of pure cultures. The pure cultures were then transferred to two 96-well LB liquid culture plates containing 0.1% L-tryptophan and incubated at 30°Cfor 2 days. Subsequently, an equal volume of Salkowski reagent (prepared from 50 mL of 35% perchloric acid and 1 mL of 0.5 M FeCl_3_ solution) was added to one of the plates, and after 30 min of color development in the dark, the appearance of a pink coloration indicated the ability to produce IAA. Cultures from corresponding wells with deeper coloration on the other plate were selected for streak purification and then incubated at 30°C for 3 days. Single colonies were picked and isolated by streaking, and stored at −80°C for preservation.

### 2.2 Salt tolerance and plant growth-promoting function test of primary screened strains

Primary Screened strains were inoculated on LB solid medium containing 4, 6, 8, 10, and 12% NaCl to test the NaCl tolerance of strains. The ability of the strains to produce IAA was evaluated using a colorimetric assay (Kumar and Audipudi, 2015). Briefly, the strains were inoculated into LB liquid mediums containing 0.1% L-tryptophan and then incubated at 30°C for 2 days. After centrifugation at 6000 rpm for 10 min, 2 mL of the supernatant was mixed with 2 mL of Salkowski reagent, and color development was observed for 30 min. Absorbance was measured at 530 nm and IAA content was determined based on a standard curve. The ability of the bacterial isolates to solubilize phosphate was tested using the plate assay method (Nautiyal, 1999; Amri et al., 2023). After inoculating fresh bacterial suspension on PVK and NBRIP agar media and incubating at 30°C for 3 days, the appearance of a solubilization halo around the colonies indicated phosphate solubilization capability; the diameters of the strains and solubilization halos were recorded. Isolates were inoculated onto Ashby medium and considered to have nitrogen-fixing ability if they could still grow on Ashby medium after three successive transfers. All the above tests were repeated three times.

### 2.3 Morphological observation and molecular identification of dominant strains

The dominant bacterial strains isolated above were activated on LB solid mediums and incubated at 30°C for 48 h, with continuous observations and recordings of colony color, shape, margin, elevation, and surface characteristics. Gram staining was performed according to the instructions of the Biosharp Gram Staining Kit. Bacterial DNA was extracted using a bacterial DNA extraction kit. The 16S rDNA sequence fragments of the strains were amplified using the universal primers 27F (5’-AGAGTTTGATCMTGGCTCAG-3′) and 1492R (5’-TACGGYTACCTTGTTACGACTT-3′). The total PCR reaction system was 50 μL, including 2× PCR Taq Mix 25 μL, template DNA 1.0 μL, primer 27F (10μmmol/L) 1 μL, primer 1492R (10 μmmol/L) 1 μL, BSA 1 μL, and ddH_2_O 21 μL. The PCR thermal cycling conditions were 95°C for 2 min for initial denaturation, followed by 31 cycles of 95°C for 15 s (denaturation), 53°C for 15 s (annealing), 72°C for 15 s (extension), and a final extension at 72°C for 5 min. Products were checked by 1.5% agarose electrophoresis. Samples were then sent to Guangdong Meige Gene Technology Co., Ltd. for Sanger sequencing. The obtained 16S rRNA gene sequences were analyzed using the EzTaxon^1^ online database. A phylogenetic tree was constructed using the Maximum Likelihood Estimate method in MEGA 11.0 software, calculating evolutionary distances with the General Time Reversible (GTR) model, with bootstrap testing repeated 500 times to determine the taxonomic status of the isolated strains.

### 2.4 Preparation of the bacterial inoculum

According to the growth-promoting function and identification results, 3 strains of *Bacillaceae* were selected for the pot experiment, which was P2, S37, and S40, respectively. After the above spare strains were prepared by growing every strain in LB at 28°C for 48 h, the cultures were harvested and diluted with sterile normal saline (0.9% NaCl) to a final OD of 1.0 at 600 nm (~ 10^9^ CFU mL^−1^), and bacterial inoculums were prepared.

### 2.5 Design and treatments of experiment, and plant growth conditions

Uniformly sized *Reaumuria soongorica* seeds, harvested in 2021 from Qingtuhu in Minqin County, were selected and disinfected with a 1% sodium hypochlorite solution for 30 min, then rinsed five times with sterile water. In April 2023, the seeds were sown in trays. The seedlings were transplanted into plastic flower pots with a diameter of 24.8 cm and a height of 28 cm, with one plant per pot, when they reached a height of 5 cm. The plastic flower pots contained 5 kg of a substrate mixture of soil, sandy soil, and humus (1,2,1), totaling 100 pots. After acclimatization of the seedlings, those with uniform growth were selected for treatment. The experiment included a control (CK, no NaCl or growth-promoting bacteria added), NaCl treatments (S, only 400 mM NaCl added), and treatments with the three strains P2, S37, and S40 alone, as well as combined treatments of NaCl and strains (P2 + S, S37 + S, S40 + S). Each treatment had six replicates; three replicates were used for biomass measurement, and three for physiological and metabolite measurements. Previous research by our team indicated that *Reaumuria soongorica* seedlings could survive under 400 and 500 mM NaCl stress, but growth was severely inhibited (Yan et al., 2022). NaCl is one of the main salts in saline-alkali soils (Chen et al., 2021). Therefore, this study selected 400 mM NaCl for salt stress treatment. To avoid osmotic shock, each pot was watered with 500 mL of NaCl solution per day for 3 consecutive days, reaching a total volume of 1,500 mL; controls and single-strain treatments were watered with an equal volume of water. Two days after the NaCl stress treatment, 100 mL of the bacterial inoculum was applied to the root area of the plants, while controls were inoculated with an equal volume of sterile physiological saline. Inoculation was repeated every 15 days using the same methodology for a total of three times. *Reaumuria soongorica* seedlings grew under natural conditions at the experimental site of Gansu Agricultural University, located in Anning District, Lanzhou City, Gansu Province (36°5′N, 103°42′E), which is in a mid-temperate climate zone with distinct inland climate characteristics, clear seasonal changes, ample sunlight, and dry weather.

### 2.6 Growth index measurement of *Reaumuria soongorica* seedlings

Sampling was conducted 56 days after treatment of the *Reaumuria soongorica* seedlings. Photographs were taken with a digital camera before sampling, and a ruler was used to measure the height of the seedlings; then, destructive sampling was carried out, collecting the aboveground and underground parts of the plants and bringing them back to the laboratory in a low-temperature sampling box, washed with pure water, and surface moisture was blotted with filter paper before weighing the fresh weight. The total root length was determined using the LA-S root analysis system (WSEEN, China).

### 2.7 Physiological index measurement of *Reaumuria soongorica* seedlings

A sample of 0.3 g fresh leaf tissue was placed in a 2 mL centrifuge tube, along with a small amount of quartz sand, and 600 mL of pre-cooled extract solution was added along with 20 zirconium oxide beads. The sample was ground for 5 min at 30 Hz using a Tissue Grinde (DROIDE, China), the homogenate was then transferred to a 10 mL centrifuge tube, and the 2 mL centrifuge tube was rinsed three times with 4 mL of extract solution to 10 mL centrifuge tube. The content of chlorophyll a, chlorophyll b, total chlorophyll, and carotenoids were measured using spectrophotometry (Li, 2000). Malondialdehyde (MDA) content was determined using the thiobarbituric acid colorimetric method (Li, 2000); proline content was measured using the acid ninhydrin method; soluble sugar content was assessed using the anthrone colorimetric method (Li, 2000); and soluble protein content was determined using the Coomassie Brilliant Blue G-250 method (Li, 2000). Na^+^ and K^+^ content was measured using the flame photometric method (Fang et al., 2018).

### 2.8 Widely targeted metabolome analysis using LC–MS/MS

The leaves of *Reaumuria soongorica*, treated for a duration of 56 days, were collected as samples for widely targeted metabolomics analysis. Three biological replicates were taken, and all samples were immediately frozen in liquid nitrogen and stored at −80°C. The S40 strain with the most effective growth promotion was chosen for metabonomics analysis. The freeze-dried samples were crushed and extracted using the procedure reported by Meng et al. (2022). The quality control (QC) sample was prepared by mixing an equal aliquot of the supernatants from all of the samples to evaluate the reproducibility and stability of the whole LC–MS/MS analysis. The sample analysis was performed using a UHPLC system (Sciex, United States) with an ACQUITY UPLC HSS T3 column (1.8 μm 2.1 × 100 mm, Waters, United States), coupled to a QTrap 6,500+ Mass Spectrometer (Sciex, United States). Metabolome raw data collected by LC–MS/MS were processed using SIMCA software (Version 16.0.2), including peak extraction, retention time, peak area, logarithmic transform, etc. Differential metabolites (DEMs) were screened based on *p*-value <0.05 among the metabolites with variable importance in projection (VIP) >1.0. In addition, the metabolic pathway enrichment of differential metabolites was analyzed by using KEGG^2^ databases. Metabolome analysis was conducted by Shanghai Biotree Biomedical Technology Company (Shanghai, China).

### 2.9 Statistical analysis

All experiments were designed with three replicates and analyzed using SPSS 26.0 software for one-way analysis of variance (ANOVA). Duncan’s multiple range test was used for significant difference comparisons between groups (α = 0.05), and graphs were created using Origin 2021 and Adobe Illustrator 2022 software.

## 3 Results

### 3.1 Screening and identification results of PGPR strains

#### 3.1.1 Tolerance to NaCl stress and growth-promoting activity of dominant strains

A total of 142 bacterial strains were isolated from the rhizosphere of *Reaumuria soongorica* in the desert regions of the Northwest. These strains exhibited various growth-promoting functions, including the capability of producing IAA, fixing nitrogen, and solubilizing organic and inorganic phosphorus. For further study, this research selected strains P2, S37, and S40, which not only have multiple growth-promoting functions but also can tolerate high levels of NaCl stress, up to 8%. Specific NaCl tolerance concentrations and growth-promoting functions are shown in Table 1.

**Table 1:** NaCl tolerance concentrations and the growth promoting function of the dominant strains.

Name	E.coli	P2	S37	S40
Maximal tolerable dose of NaCl (%)	8%	12%	8%	12%
IAA (μg mL-1)	20.66 ± 0.88^d^	50.91 ± 1.36^c^	80.39 ± 1.54^a^	61.99 ± 1.19^b^
Nitrogen fixation	–	***	***	***
Inorganic phosphorus solubilization (D/d)	–	1.35 ± 0.07^a^	1.30 ± 0.04^a^	1.39 ± 0.13^a^
Organic phosphorus solubilization (D/d)	–	2.72 ± 0.10^a^	1.78 ± 0.08^b^	1.63 ± 0.15^b^

D/d, diameter halo/diameter colony; IAA, indole acetic acid; +, presence of activity; −, absence of activity; Mean values labeled with the same superscript letter within the same line were not significantly different at *p* < 0.05 following Duncan’s test; *E.coli*, Control strain *Escherichia coli*.

#### 3.1.2 Morphological observation and gram staining results of dominant strains

**Figure 1:**
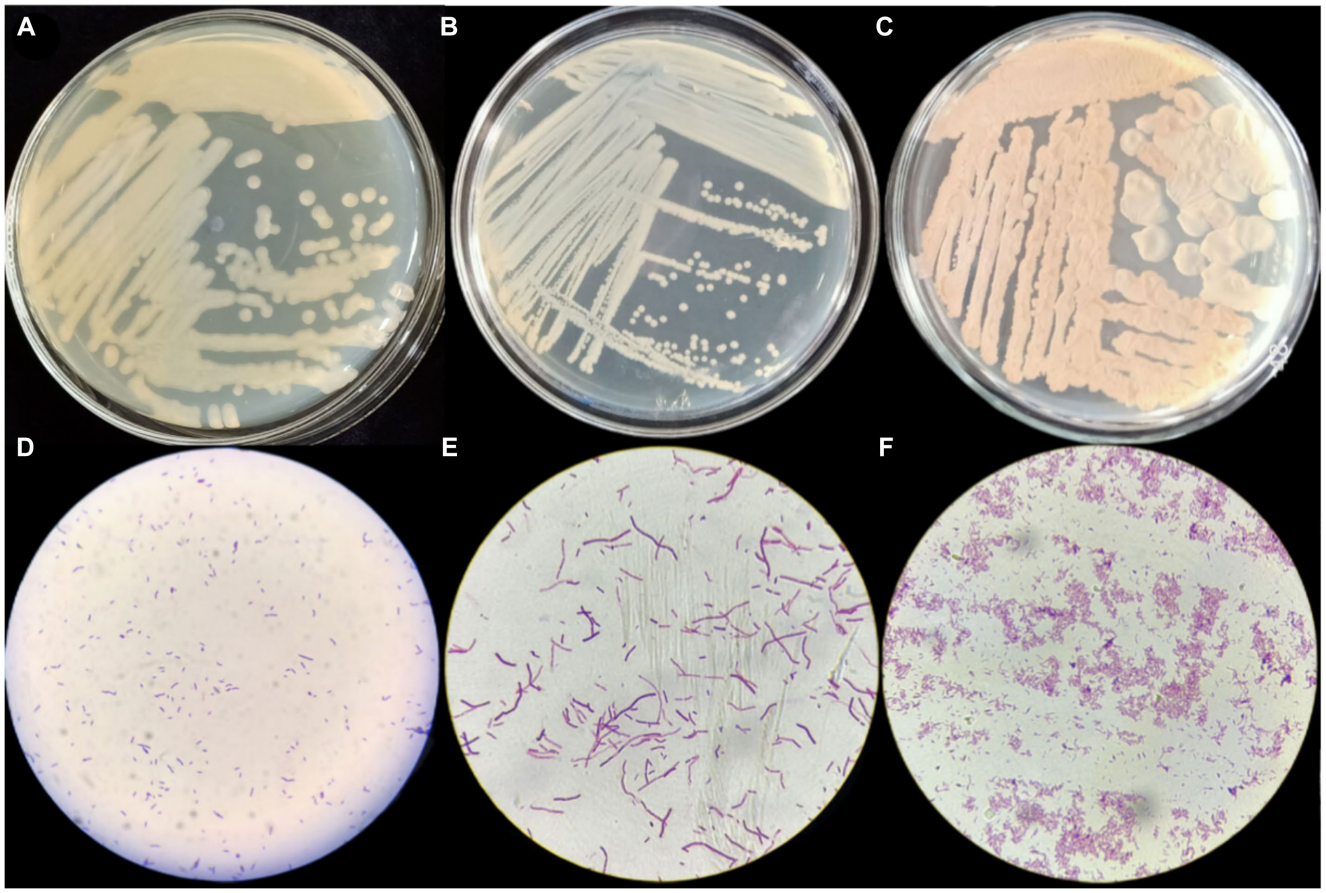
Morphological characteristics and Gram staining results of strains P2, S37, and S40. **(A–C)** The morphological structure diagrams of strains P2, S37, and S40; **(D–F)** the 100x microscope images of Gram’s stain of strains P2, S37, and S40.

Gram staining of strains P2, S37, and S40 showed positive results, with short rod-shaped cells with blunt ends, occurring in pairs or chains. On LB agar plates, the colonies were yellow, with smooth, moist surfaces and irregular edges. The colonies were opaque with uneven margins (see Figure 1), pre and preliminarily identified as *Bacillus*.

#### 3.1.3 Molecular identification results of dominant strains

**Figure 2:**
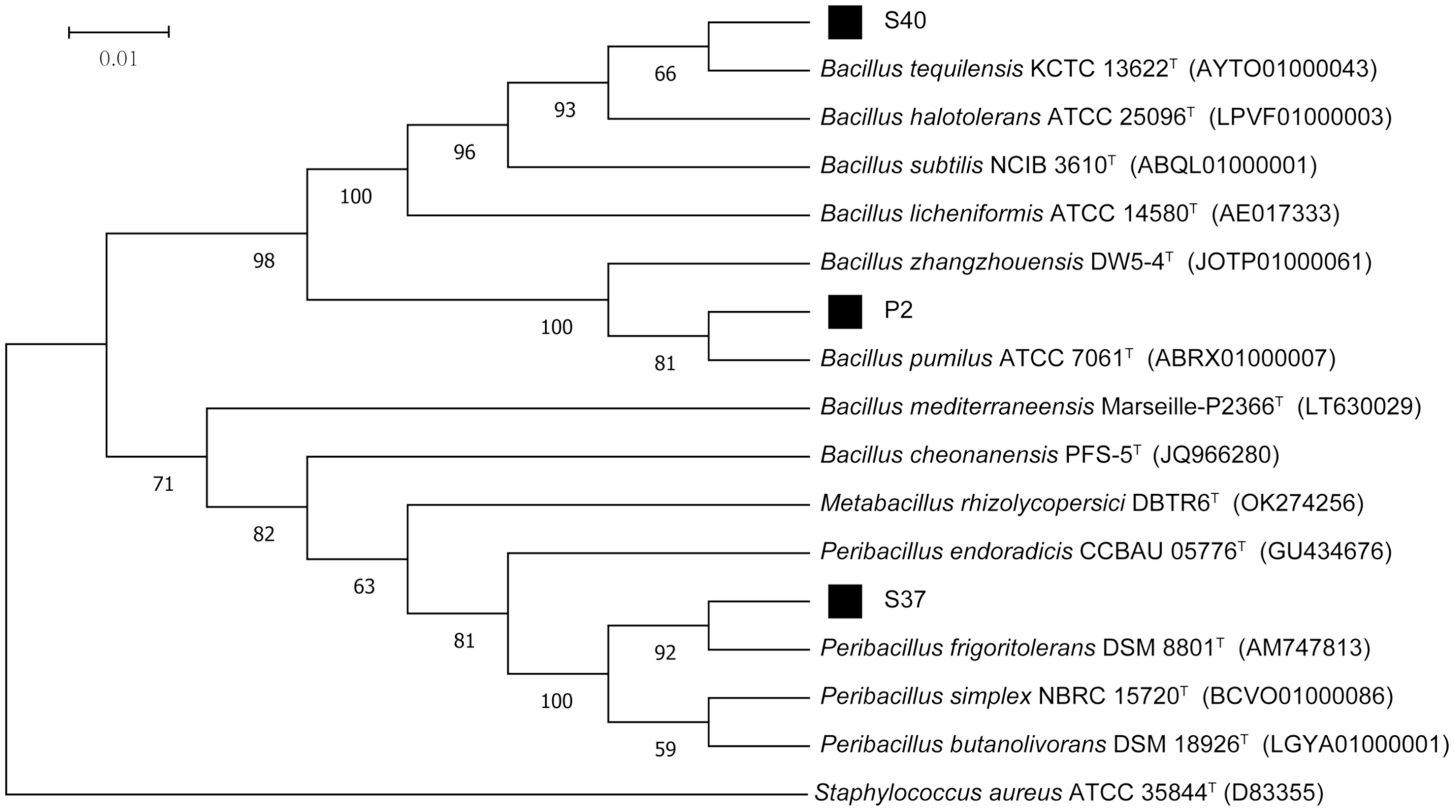
The phylogenetic tree of strains P2, S37, and S40 based on 16S rDNA sequencing results. *Staphylococcus aureus* ATCC 35844^T^ is an outgroup; Superscript T are type strains; the scale length is 1% base difference.

The 16S rDNA sequencing results of strains P2, S37, and S40 were compared and analyzed. The phylogenetic tree based on the 16S rRNA gene sequences, as shown in Figure 2, indicated that the dominant strains P2 and S40 belong to the *Bacillus*, and S37 belongs to the *Peribacillus.* which was consistent with morphological identification results. In addition, the gene fragment of strain P2 was 1,385 bp in length, which was the same branch as *Bacillus pumilus* KCTC 13622^T^ and had a similarity of 99.93%. The gene sequence length of strain S37 was 1,388 bp, belonging to the same branch as *Peribacillus frigoritolerans* DSM 8801^T^, with a similarity of 100%. The gene fragment of strain S40 was 1,384 bp, belonging to the same branch as *Bacillus tequilensis* KCTC 13622^T^, with a similarity of 100%. So, combining morphological and molecular biological characteristics, P2, S37, and S40 could be preliminarily determined as *Bacillus pumilus, Peribacillus frigoritolerans, Bacillus tequilensis*, respectively. The 16S rDNA sequences of P2, S40, and S37 strains have been deposited in the NCBI database with GenBank accession numbers PP515614, PP515615, and PP515616, respectively.

### 3.2 *Bacillaceae* improved growth and enhanced salt tolerance of *Reaumuria soongorica* seedlings under NaCl stress

#### 3.2.1 *Bacillaceae* promoted the growth of *Reaumuria soongorica* seedlings

**Figure 3:**
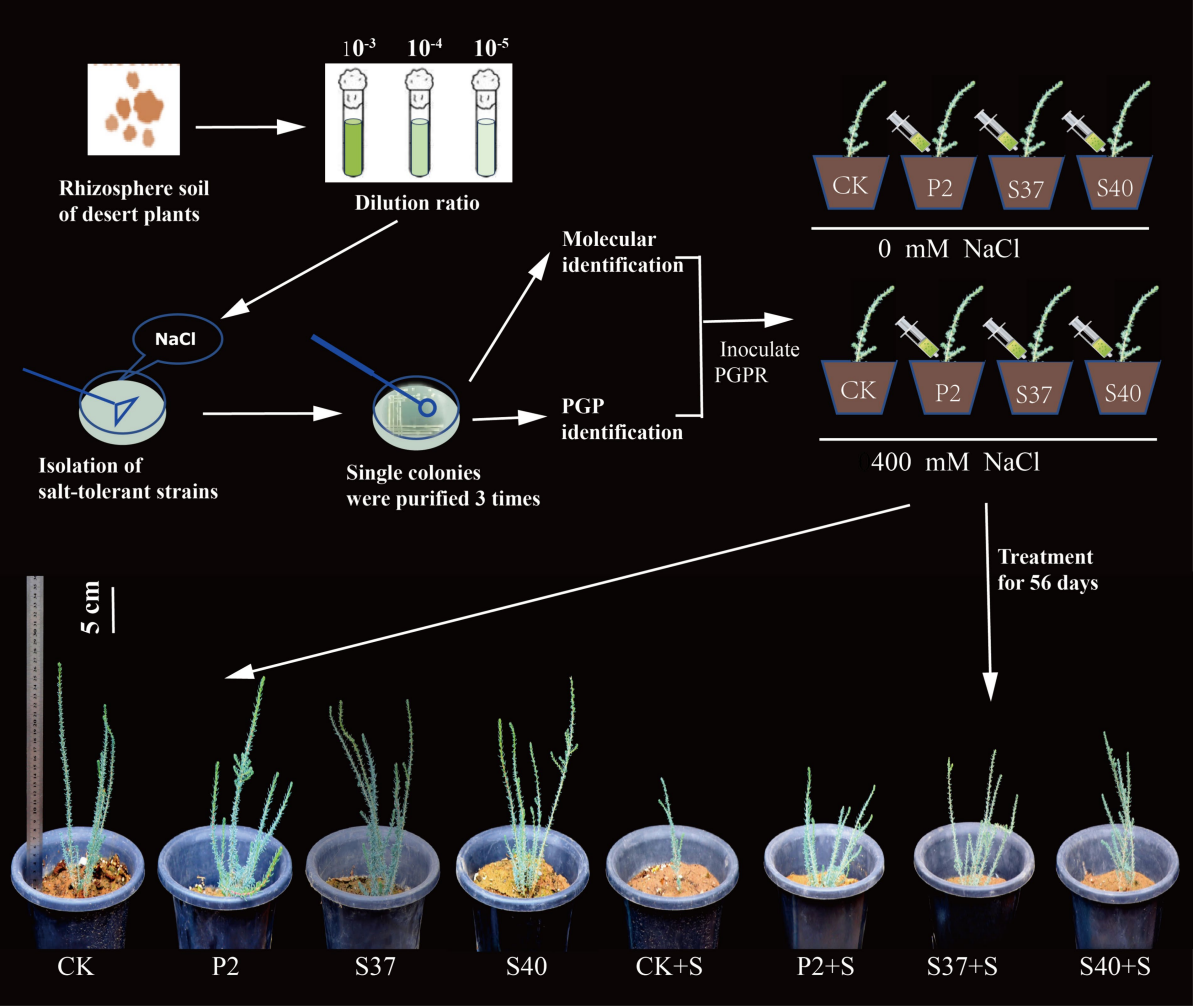
Experimental processing flowchart and the growth states of *Reaumuria soongorica* seedlings after inoculation with strains P2, S37, and S40.

**Figure 4:**
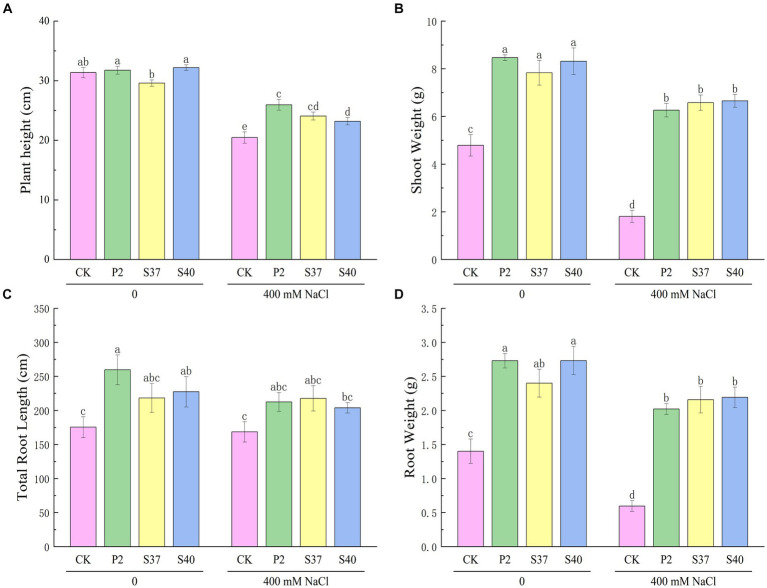
The effect of strains P2, S37, and S40 on the growth of *Reaumuria soongorica* seedlings under NaCl stress. **(A)** Plant height, **(B)** Shoot Weight, **(C)** Total Root Length, and **(D)** Root Weight. Mean values labeled with the same superscript letter within the same line were not significantly different at *p < 0.05* following Duncan’s test.

Inoculation with the three *Bacillaceae* strains had a significant impact on the growth and salt tolerance of *Reaumuria soongorica* seedlings (Figures 3, 4). Inoculation with strains P2, S37, and S40 showed no significant difference in seedling height compared to the control, but the aboveground fresh weight increased significantly by 83.17, 72.54, and 94.54% (*p* < 0.05), respectively. Inoculation also increased the total root length and fresh weight of *Reaumuria soongorica* seedlings, with P2 and S40 single-strain treatment groups showing a significant increase in total root length by 48.00 and 29.62% over the control, respectively; and P2, S37, and S40 single-strain treatment groups showing a significant increase in root fresh weight by 94.75, 68.86, and 94.98% (*p < 0.05*) over the control, respectively. NaCl stress significantly reduced the height, aboveground, and root fresh weight, and of *Reaumuria soongorica* seedlings, but inoculation with P2, S37, and S40 strains resulted in a significant increase in seedling height by 26.87, 17.59, and 13.36% (*p* < 0.05) compared to NaCl treatment, and more than doubled the aboveground and root fresh weight. Thus, inoculation with strains P2, S37, and S40 had positive effects on *Reaumuria soongorica* growth and Salt tolerance.

#### 3.2.2 Effect of *Bacillaceae* on photosynthetic pigment content in *Reaumuria soongorica* seedling leaves

Shown in Table 2, inoculation with P2, S37, and S40 strains upregulated the photosynthetic pigment content in the leaves of *Reaumuria soongorica* seedlings. Specifically, inoculation with strain S40 resulted in a significant increase in the content of chlorophyll a, b, and carotenoids by 22.95, 20.14, and 24.33% (*p* < 0.05) over the control. Under NaCl stress, the contents of chlorophyll a, b, and total chlorophyll in the leaves of *Reaumuria soongorica* seedlings were significantly reduced by 23.08, 21.48, and 22.81% compared to the control. However, after inoculation with P2, S37, and S40 strains, the four photosynthetic pigments in the leaves of *Reaumuria soongorica* seedlings were significantly higher than those in the NaCl treatment and significantly exceeded control levels. Compared to the NaCl treatment, inoculation with strain S40 significantly increased the content of chlorophyll a, b, total chlorophyll, and carotenoids in *Reaumuria soongorica* seedling leaves by 87.00, 85.47, 86.74, and 20.81%, respectively (*p < 0.05*). Thus, inoculation with strains P2, S37, and S40 increased the content of photosynthetic pigments in leaves of *Reaumuria soongorica* seedlings, and the effect was more obvious under NaCl stress.

**Table 2:** The content of photosynthetic pigments in the leaves of *Reaumuria soongorica* seedlings after inoculation with strains P2, S37, and S40.

NaCl level	Strain NO.	Chlorophyll a (mg L^−1^ FW)	Chlorophyll b (mg L^−1^ FW)	Total Chlorophyll (mg L^−1^ FW)	Carotenoids (mg·g^−1^FW)
0 mM NaCl	CK	0.52 ± 0.02^d^	0.10 ± 0.01^cd^	0.63 ± 0.04^d^	0.13 ± 0.01^b^
	P2	0.60 ± 0.04^cd^	0.13 ± 0.01^abc^	0.73 ± 0.05^c^	0.16 ± 0.01^a^
	S37	0.61 ± 0.03^c^	0.12 ± 0.00^bc^	0.73 ± 0.03^c^	0.16 ± 0.02^ab^
	S40	0.65 ± 0.04^bc^	0.12 ± 0.01^abc^	0.77 ± 0.04^bc^	0.16 ± 0.01^a^
400 mM NaCl	CK	0.40 ± 0.01^e^	0.08 ± 0.01^d^	0.49 ± 0.01^e^	0.15 ± 0.01^ab^
	P2	0.69 ± 0.02^ab^	0.14 ± 0.01^ab^	0.83 ± 0.02^ab^	0.18 ± 0.01^a^
	S37	0.72 ± 0.03^ab^	0.13 ± 0.01^ab^	0.85 ± 0.03^ab^	0.16 ± 0.01^a^
	S40	0.75 ± 0.01^a^	0.15 ± 0.01^a^	0.89 ± 0.02^a^	0.18 ± 0.00^a^

Mean values labeled with the same superscript letter within the same line were not significantly different at *p* < 0.05 following Duncan’s test.

#### 3.2.3 Effect of *Bacillaceae* on osmotic adjustment substances content in *Reaumuria soongorica* seedling leaves

Inoculation with P2, S37, and S40 strains significantly affected the contents of osmotic substances in *Reaumuria soongorica* seedlings (Table 3). The proline and soluble protein content in the leaves of *Reaumuria soongorica* seedlings inoculated with strains P2, S37, and S40 were significantly higher than the control, with strain S40 showing a significant increase of 38.60 and 26.12%, respectively; but, soluble sugar content in the leaves of *Reaumuria soongorica* seedlings significantly decreased after inoculated with P2, S37, and S40 strains, by 13.66, 13.77, and 13.83%, respectively (*p < 0.05*). Under NaCl stress, the proline content in the leaves of *Reaumuria soongorica* seedlings inoculated with P2, S37, and S40 strains showed no significant change, while the soluble protein content was significantly increased by 19.98, 23.57, and 24.25% (*p < 0.05*) compared to the NaCl treatment. However, compared to the NaCl treatment, the soluble sugar content in the leaves of *Reaumuria soongorica* seedlings stress significantly decreased by 10.44, 10.51, and 10.48%, respectively (*p < 0.05*). Overall, inoculation with strains P2, S37, and S40 regulated the contents of osmoprotectants in leaves of *Reaumuria soongorica* seedlings, maintaining ion homeostasis, and preventing oxidative damage.

**Table 3:** The content of osmotic adjustment substances and MDA contents in the leaves of *Reaumuria soongorica* seedlings after inoculation with strains P2, S37, and S40.

NaCl level	Strain NO.	Proline content (μg·g^−1^FW)	Soluble protein content (μg·g^−1^FW)	Soluble sugar content (mg·g^−1^FW)	MDA content (μmol·g-1FW)
0 mM NaCl	CK	100.35 ± 5.83^b^	6.20 ± 0.34^c^	14.35 ± 0.32^a^	3.65 ± 0.27^d^
	P2	153.07 ± 10.57^a^	7.98 ± 0.44^b^	9.96 ± 0.26^b^	2.61 ± 0.19^e^
	S37	136.11 ± 7.04^a^	7.68 ± 0.09^b^	8.22 ± 0.59^c^	3.10 ± 0.16^e^
	S40	139.08 ± 8.53^a^	7.81 ± 0.49^b^	7.42 ± 0.11^c^	2.76 ± 0.16^e^
400 mM NaCl	CK	150.49 ± 6.72^a^	7.68 ± 0.33^b^	11.16 ± 0.20^b^	6.44 ± 0.08^a^
	P2	140.55 ± 11.05^a^	9.21 ± 0.18^a^	8.03 ± 0.73^c^	4.80 ± 0.24^b^
	S37	132.90 ± 4.22^a^	9.49 ± 0.16^a^	7.26 ± 0.47^c^	4.47 ± 0.13^bc^
	S40	135.44 ± 6.84^a^	9.54 ± 0.20^a^	7.54 ± 0.39^c^	4.19 ± 0.14^c^

Mean values labeled with the same superscript letter within the same line were not significantly different at *p* < 0.05 following Duncan’s test.

#### 3.2.4 Effect of *Bacillaceae* on malondialdehyde content in *Reaumuria soongorica* seedling

Malondialdehyde (MDA) is one of the most common indicators used to detect lipid peroxidation in cells and tissues. Inoculation with P2, S37, and S40 strains, the MDA content in the leaves of *Reaumuria soongorica* seedlings were significantly reduced by 28.54, 15.14, and 24.31% (*p < 0.05*) compared to the control; and under NaCl stress, the MDA contents were significantly reduced by 25.41, 30.62, and 34.85% (*p < 0.05*) compared to the NaCl treatment (Table 3). Thus, inoculation with strains P2, S37, and S40 had positive effects on preventing oxidative damage of *Reaumuria soongorica* seedlings and preserving the cell membrane structure.

#### 3.2.5 Effect of *Bacillaceae* on Na^+^, K^+^ content in *Reaumuria soongorica* seedling

Under NaCl stress, inoculation with P2, S37, and S40 strains, Na^+^, K^+^ contents, and the Na^+^- K^+^ ratio were significantly downregulated (Table 4). Inoculation with strains P2, S37, and S40 showed no significant difference in Na^+^, K^+^ contents, and the Na^+^- K^+^ ratio compared to the control. Under NaCl stress, Na^+^, K^+^ contents, and the Na^+^- K^+^ ratio in the leaves of *Reaumuria soongorica* seedlings were significantly increased by 173.90, 23.45, and 121.81%, respectively (*p < 0.05*) compared to the control. However, after inoculation with P2, S37, and S40 strains, Na^+^ contents in the leaves of *Reaumuria soongorica* seedlings were significantly decreased by 35.80, 30.59, and 35.92%, respectively (*p < 0.05*) compared to the NaCl treatment; K^+^ contents were significantly decreased by 21.13, 17.07, and 25.57%, respectively (*p < 0.05*); the Na^+^- K^+^ ratio was significantly decreased by 18.47, 16.13, and 13.91%, respectively (*p < 0.05*). The results showed that inoculation with strains P2, S37, and S40 decreased Na^+^ content and Na^+^- K^+^ ratio in leaves of *Reaumuria soongorica* seedlings under NaCl stress to re-establish ion balance, and alleviated the toxicity of salt stress.

**Table 4:** The content of Na^+^, K^+^ contents, and the Na^+^- K^+^ ratio in the leaves of *Reaumuria soongorica* seedlings after inoculation with strains P2, S37, and S40.

NaCl level	Strain NO.	Na^+^ content (mg·g^−1^ DW)	K^+^ content (mg·g^−1^ DW)	Na^+^/K^+^ ratio
0 mM NaCl	CK	32.91 ± 1.04^c^	15.03 ± 0.21^bc^	2.19 ± 0.07^c^
	P2	39.33 ± 0.84^c^	16.47 ± 0.62^b^	2.39 ± 0.14^c^
	S37	37.61 ± 1.18^c^	15.37 ± 0.57^bc^	2.45 ± 0.01^c^
	S40	34.53 ± 0.90^c^	15.1 ± 0.52^bc^	2.29 ± 0.14^c^
400 mM NaCl	CK	90.14 ± 3.47^a^	18.56 ± 0.26^a^	4.86 ± 0.12^a^
	P2	57.87 ± 2.78^b^	14.64 ± 0.87^c^	3.96 ± 0.21^b^
	S37	62.56 ± 2.18^b^	15.39 ± 0.82^bc^	4.07 ± 0.26^b^
	S40	57.76 ± 4.58^b^	13.81 ± 0.58^c^	4.18 ± 0.25^b^

Mean values labeled with the same superscript letter within the same line were not significantly different at *p* < 0.05 following Duncan’s test.

### 3.3 Effect of *Bacillus* S40 on metabolites in *Reaumuria soongorica* seedling leaves under NaCl stress

#### 3.3.1 Classification of metabolites

Based on the above results, inoculated with P2 and S37 strains, the growth indexes such as plant height, total root length, biomass, and physiological indexes such as photosynthetic pigment and osmotic adjustment substance MDA of Reaumuria soongorica seedlings under salt stress showed the same trend as the S40 strain. S40 strain had the best effect of promoting growth. Therefore, only the S40 strain was selected for metabolism analysis in this study. After filtering and normalizing the raw data of the targeted metabolome, a total of 400 metabolites were obtained, including 59 flavonoids, 54 alkaloids, 49 phenolic compounds, 46 terpenoids, 26 lipids, 24 lignans and coumarins, 23 organic acids and derivatives, 22 amino acids and derivatives, 19 phenylpropanoids, 16 carbohydrates and alcohols, 14 plant hormones, 10 nucleosides and derivatives, 8 quinones, 7 aromatic compounds, 4 organic acids and derivatives, 2 vitamins, and 26 other metabolites (Supplementary Table S1).

#### 3.3.2 Differential analysis of metabolites

**Figure 5:**
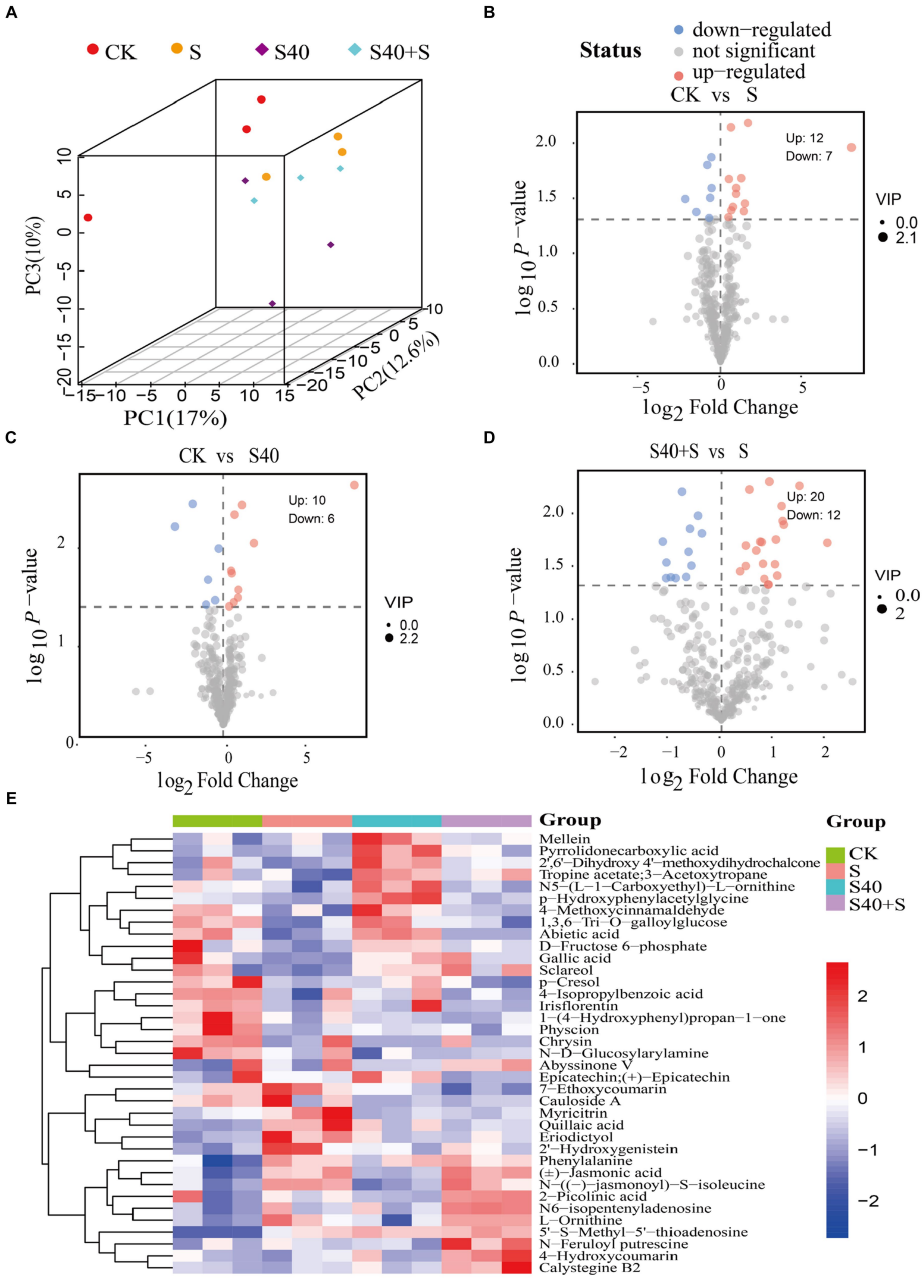
Differential metabolites in leaves of *Reaumuria soongorica* seedlings. **(A)** Score scatter plot 3D of PCA model. **(B–D)** The volcano maps between different groups, each dot represented a metabolite, the x-axis represents the fold change of the comparison of various substances (taking the logarithm of base 2), the y-axis represents the negative logarithm of *p*-value (taking the negative logarithm of base 10). **(E)** Hierarchical clustering heatmap of differential metabolites, red indicates that the substance is highly expressed in the group where it is located, and blue indicates that the substance is low in the group where it is located.

Based on the *p*-value <0.05 among the metabolites with variable importance in projection (VIP) >1.0, 37 differential metabolites (DEMs) were screened (Supplementary Table S2). In the 3D PCA plot, biological replicates for each of the four different treatments clustered together in different areas, suggesting that there were significant differences in metabolites (Figure 5A). Volcano plots (Figures 5B–D) exhibited trends in differential metabolite changes between different treatment groups (*p* < 0.05). There were 19 differential metabolites (12 upregulated, 7 downregulated) between the control (CK) and S treatment, with significant upregulation of 5’-S-Methyl-5′-thioadenosine, N-((−)-jasmonoyl)-S-isoleucine, L-Ornithine, eriodictyol, and 2′-hydroxygenistein (log_2_(Fold Change) > 1), and significant downregulation of amygdalin, and physcion (log_2_(Fold Change) < 0.5); 16 differential metabolites (10 upregulated, 6 downregulated) between the control (CK) and S40 treatment, with significant upregulation of 5’-S-Methyl-5′-thioadenosine, pyrrolidone carboxylic acid, and p-hydroxyphenylacetylglycine (log_2_(Fold Change) > 1), and significant downregulation of chrysin, cauloside A, and N-D-Glucosylarylamine (log_2_(Fold Change) < 0.5); 32 differential metabolites (20 upregulated, 12 downregulated) between S40 + S and S treatment, with significant upregulation of eicosenoic acid, talatisamine, 7-ethoxycoumarin, dihydrokavain, epicatechin, quillaic acid, 3-hydroxy-4-methoxycinnamic acid, and epicatechin gallate (log_2_(Fold Change) > 1), and significant downregulation of 4-hydroxycoumarin, sclareol, and alpha-hexylcinnamaldehyde (log_2_(Fold Change) < 0.5). The heatmap of 37 differential metabolites analysis for different treatment samples was shown in Figure 5E, and hierarchical clustering analysis was performed on the differential metabolites. There was an obvious separation in metabolites between the control (CK) and the NaCl treatments (S), the control (CK) and S40, and S40 and S40 + S treatments. Overall, there were significant differences in metabolites of leaves *Reaumuria soongorica* seedlings in different treatments.

#### 3.3.3 KEGG enrichment analysis of differential metabolites

**Figure 6:**
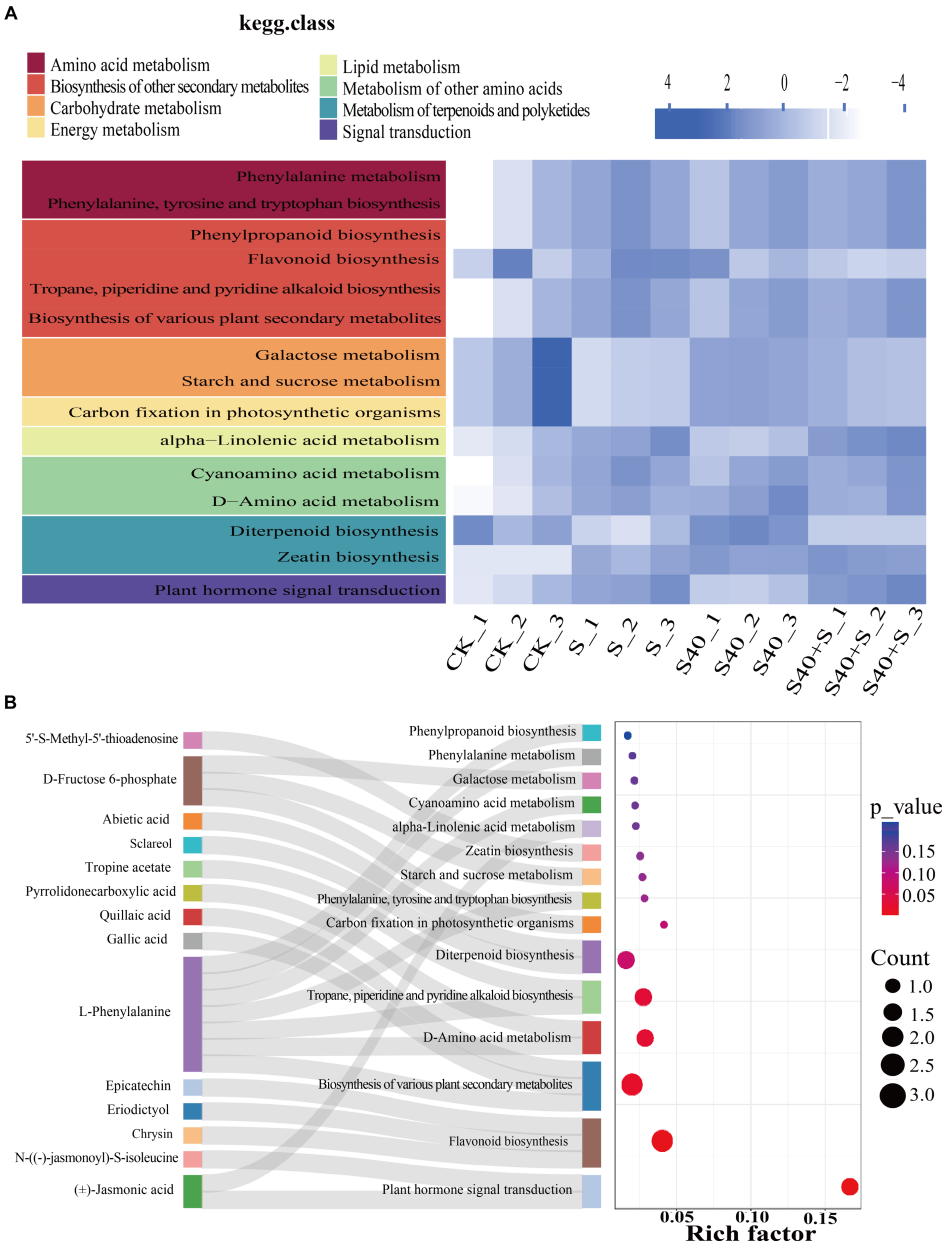
Enrichment analysis of top fifteen KEGG metabolic pathways of differential metabolites in leaves of *Reaumuria soongorica* seedlings. **(A)** KEGG heatmap for different groups, with color blocks representing the relative content of all differential metabolites annotated in the corresponding positional pathway. **(B)** Sankey bubble combination diagram, with the horizontal axis representing the ratio of the number of differential metabolites in this pathway to the number of metabolites annotated in this pathway. The redder the bubbles, the more significant the enrichment of differential metabolites in this pathway; the larger the bubble, the more differential metabolites there are.

Using Arabidopsis as a model organism, differential metabolites were mapped to KEGG metabolic pathways for pathway and enrichment analyses. KEGG heatmap based on the relative abundance of differential metabolites and the classification of pathways was shown in Figure 6A, which displays the total amount of annotated differential metabolites in a certain KEGG metabolic pathway in different samples. Under NaCl stress, the total amount of N-((−)-jasmonoyl)-S-isoleucine, (J)-Jasmonic acid, and 5’-S-Methyl-5′-thioadenosine were significantly up-regulated compared with the control (CK) (*p* < 0.05), and they were annotated to plant hormone signal transduction and zeatin biosynthesis pathway, while the amount of D-fructose 6-phosphate was significantly down-regulated compared with the control (CK) (*p* < 0.05), and it was annotated to carbon fixation in photosynthetic organisms, starch and sucrose metabolism and galactose metabolism pathway. However, inoculation with the S40 strain, the amount of D-fructose 6-phosphate was significantly up-regulated compared with the NaCl treatments (S) (*p* < 0.05), while the amount of epicatechin, chrysin, eriodictyol, and sclareol were significantly down-regulated compared with NaCl treatments (S) (*p* < 0.05), and they were annotated to flavonoid biosynthesis and diterpenoid biosynthesis pathway. The Sankey bubble diagram (Figure 6B) shows the enrichment analysis of the top fifteen KEGG metabolic pathways of differential metabolites in leaves of *Reaumuria soongorica* seedlings. Five pathways were significantly enriched, including plant hormone signal transduction, flavonoid biosynthesis, biosynthesis of various plant secondary metabolites, D-amino acid metabolism, and tropane, piperidine, and pyridine alkaloids biosynthesis (*p* < 0.05). L-phenylalanine is involved in the biosynthesis of various plant secondary metabolites, D-amino acid metabolism, tropane, piperidine, and pyridine alkaloids biosynthesis, the biosynthesis of phenylalanine, tyrosine, and tryptophan, cyanogenic amino acids, phenylalanine metabolism, and phenylpropanoid biosynthesis. These results indicate that inoculation with strain S40 induces plant metabolome reprogramming to regulate cell metabolism, including plant hormone signal transduction and phenylalanine, tyrosine, and tryptophan biosynthesis pathways.

## 4 Discussion

**Figure 7:**
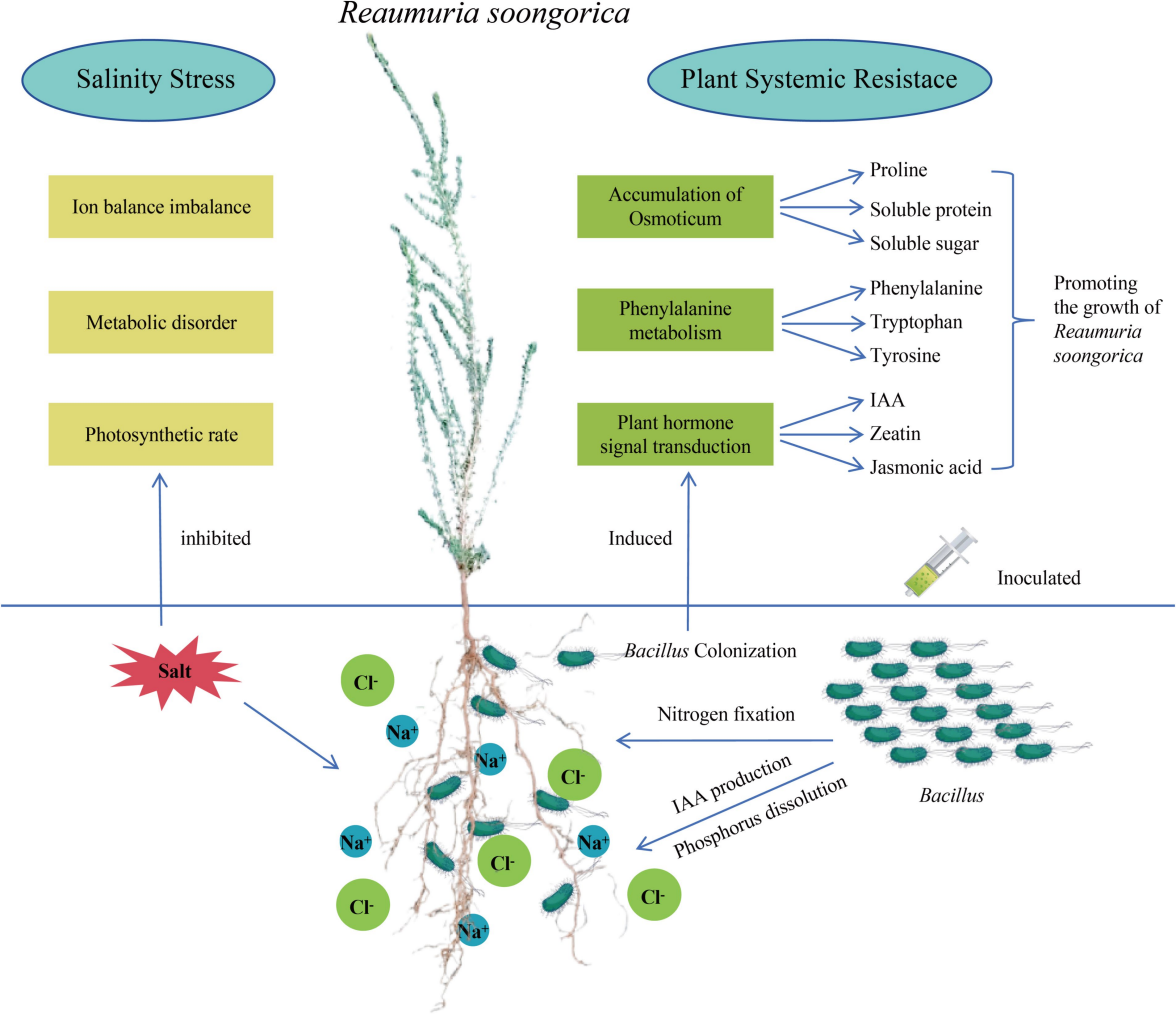
Pattern diagram of *Bacillus subtilis* promoting the growth of *Reaumuria soongorica* seedlings and enhancing salt tolerance mechanism.

Desert plants live in environments with various abiotic stresses such as nutrient deficiency, drought, and salinization. PGPR can help plants cope with adversity in various ways; however, whether it is a single isolate or SynCum, it must be adapted to the target environment to exert its growth-promoting function (Saad et al., 2020; Schmitz et al., 2022). Therefore, this study isolated and screened PGPR with various growth-promoting functions and salt resistance from the rhizosphere of the desert plant *Reaumuria soongorica* using high-throughput bacterial culture and identification methods. Through molecular identification, the dominant strains were identified as belonging to the *Bacillaceae*. This study found that inoculation with strains P2, S37, and S40 significantly improved growth indicators such as plant height, total root length, and biomass, and notably increased salt stress tolerance in *Reaumuria soongorica* seedlings. This increase is attributed to PGPR’s ability to regulate plant photosynthetic pigment synthesis and improve photosynthesis efficiency, as well as to promote plant nutrient absorption through phosphate solubilization and nitrogen fixation. Inoculation with strain S40 can relieve salt stress in plants by regulating the synthesis of primary metabolites such as phenylalanine, tyrosine, proline, and secondary metabolites such as IAA, cytokinins, and jasmonic acid (Sunita et al., 2020; Koza et al., 2022; Liu et al., 2023). The *Bacillus* and *Peribacillus* strains screened in this study promote the growth of *Reaumuria soongorica* and enhance its salt stress tolerance by inducing systemic tolerance in the plant. A schematic diagram summarizing the mechanisms by which *Bacillaceae* promotes the growth of *Reaumuria soongorica* seedlings and enhances resistance to salt stress is presented in Figure 7.

### 4.1 *Bacillaceae* plays a significant role in promoting healthy plant growth

*Bacillus*, widely studied as a Plant Growth-Promoting Rhizobacteria (PGPR), was known for its biological control and growth promoting effects. Studies have found that *Bacillus licheniformis* and *Bacillus velezensis* promote the growth of rice (Devi et al., 2023); *Bacillus megaterium* induces systemic resistance-related metabolites to promote the growth of Arabidopsis (Liu et al., 2023); *Bacillus paralicheniformis* enhances cotton growth by significantly increasing root length and the content of brassinosteroids (Xu et al., 2023). In this study, three PGPR strains were isolated and identified as *Bacillus pumilus*, *Peribacillus frigoritolerans*, and *Bacillus tequilensis*. These PGPR strains not only produce IAA and have the ability to solubilize phosphate and fix nitrogen—characteristics of ideal PGPR—but also play a crucial role in promoting the growth of desert plants and enhancing their stress resistance, especially in barren desert environments. Pot experiments showed that under NaCl stress, the height, total root length, and fresh weight of *Reaumuria soongorica* seedlings were significantly reduced compared to the control, but inoculation with strains P2, S37, and S40 significantly increased the shoot and root fresh weight of *Reaumuria soongorica* seedlings compared to the NaCl treatment. This indicates that inoculation with *Bacillaceae* has positive effects on *Reaumuria soongorica* growth and salt tolerance.

### 4.2 *Bacillaceae* promotes plant growth and alleviates salt stress by regulating photosynthesis

In higher plants, chlorophyll a, chlorophyll b, and carotenoids are the most critical photosynthetic pigments and are essential for plant photosynthesis. Under salt stress, plants may experience disturbances in leaf area and stomatal conductance, leading to insufficient water and mineral nutrient absorption, resulting in malnutrition; excess salt can lead to chloroplast degradation, block the biosynthesis of chlorophyll and carotenoids, reduce photosynthetic efficiency, and affect plant growth (Kibria et al., 2017; Sunita et al., 2020; Kumar et al., 2021). However, PGPR can produce IAA, siderophores, and hydrolytic enzymes, and can solubilize phosphates, thereby improving chlorophyll content and membrane integrity (Jalmi and Sinha, 2022). Cherry tomato plants inoculated with PGPR have higher chlorophyll content in their leaves than uninoculated plants (El-Beltagi et al., 2022). In this study, chlorophyll a, b, and total chlorophyll in *Reaumuria soongorica* seedling leaves under NaCl stress were significantly reduced compared to the control, consistent with the previous research results (Yan et al., 2022). After inoculation with strains P2, S37, and S40, chlorophyll a, b, and carotenoids in *Reaumuria soongorica* seedling leaves under NaCl stress were significantly higher than those treated with NaCl and significantly higher than control levels. Metabolomic results showed that after inoculation with strain S40, the content of glutamate, which is involved in chlorophyll synthesis, was significantly increased in *Reaumuria soongorica* seedlings, providing a material basis for the increase in chlorophyll. Strains P2, S37, and S40 enhanced the synthesis of photosynthetic pigments by promoting phytohormone production, thus improving *Reaumuria soongorica* photosynthesis and leading to a significant accumulation of *Reaumuria soongorica* biomass, consistent with previous research findings (El-Beltagi et al., 2022; Slimani et al., 2023). It indicates that inoculation with *Bacillaceae* can promote photosynthesis by increasing the content of photosynthetic pigments, thereby promoting the growth of the plant and enhancing its salt tolerance.

### 4.3 *Bacillaceae* induces the osmoprotectants to alleviate salt ion toxicities

One of the plant’s stress response mechanisms to adverse conditions is the synthesis of osmoprotectants, which increase the solute concentration inside cells, lower the water potential, and enable plants to absorb water from the environment to sustain growth. Proline is a vital substance required for plant survival under abiotic stress. As a compatible osmolyte, it can chelate salt ions to maintain internal osmotic balance and interact with proteins to increase their solubility (Selim et al., 2021). Studies have shown that *Bacillus flexus* KLBMP 4941 promotes the growth of the halophyte Suaeda salsa under salt stress by regulating the Na^+^/K^+^ homeostasis (Xiong et al., 2020). Salt-tolerant *Bacillus* KKD1 alleviates salt stress in wheat by regulating osmotic balance and ion homeostasis to protect plant growth (Wu et al., 2022). In this study, the inoculation of strains P2, S37, S40 increased the proline and soluble protein content in *Reaumuria soongorica* seedling leaves compared to the NaCl treatment, indicating that these strains can induce *Reaumuria soongorica* seedling to synthesize proline and soluble proteins, maintaining osmotic balance in plant cells under salt stress and preventing water loss, consistent with previous research findings (Slimani et al., 2023). However, the soluble sugar content in leaves of *Reaumuria soongorica* seedlings inoculated with strains P2, S37, and S40 was significantly reduced, a result inconsistent with previous studies (El-Beltagi et al., 2022), which may be due to the involvement of soluble sugars in plant metabolism under 400 mM NaCl stress, providing energy or being converted into secondary metabolites. MDA is the end product of lipid peroxidation and an important indicator of lipid peroxidation and plasma membrane damage. Inoculation with strains P2, S37, and S40 significantly reduced MDA content in *Reaumuria soongorica* seedling leaves under both NaCl stress and non-stress conditions. It indicated that inoculation with *Bacillaceae* can promote the growth of the plant and enhance its salt tolerance by regulating the accumulation of osmoprotectants, preventing oxidative damage, and preserving the integrity of the cell membrane structure.

### 4.4 *Bacillus* S40 modulates the synthesis pathways of phenylalanine, tyrosine, and tryptophan to promote plant growth and increase salt tolerance

Phenylalanine, tyrosine, and tryptophan are precursors of natural products such as alkaloids, flavonoid compounds, plant auxins, and cell wall components. Phenylalanine is catalyzed by phenylalanine ammonia-lyase to form cinnamic acid and coumaric acid, which are further hydroxylated and methoxylated to form phenylpropanoid compounds such as caffeic acid and ferulic acid, eventually leading to the synthesis of complex secondary metabolites like coumarins, lignins, and flavonoids, which are used in plant defense against various biotic and abiotic stresses (Maeda and Dudareva, 2012; Ham et al., 2022; Liu et al., 2023). In this study, inoculation with strain S40 significantly increased the content of phenylalanine in the leaves of *Reaumuria soongorica* seedlings compared to the control (*p < 0.05*) (Supplementary Table S1). Under NaCl stress, inoculation with strain S40 significantly increased the content of metabolites such as 4-hydroxycoumarin, sclareol, and alpha-hexylcinnamaldehyde in *Reaumuria soongorica* seedling compared to the NaCl treatment (Figure 5E). L-phenylalanine is involved in D-amino acid metabolism, the biosynthesis of phenylalanine, tyrosine, and tryptophan, cyanogenic amino acids, phenylalanine metabolism, phenylpropanoid biosynthesis, and other pathway (Figure 6B). Strain S40 induced the accumulation of phenylalanine in the leaves of *Reaumuria soongorica* seedlings, which are involved in the synthesis of secondary metabolites such as coumarins, and flavonoids. Studies have shown that inoculation with *Bacillus megaterium* BT22 promotes Arabidopsis growth by accumulating flavonoids and phenylpropanoid metabolites (Liu et al., 2023). *Paenibacillus polymyxa* YC0136 induces tobacco systemic resistance by stimulating phenylpropanoid metabolism under pathogen-free conditions (Liu et al., 2020). Our study also proved this point, inoculation with strain S40 increases the content of phenylalanine in *Reaumuria soongorica* seedlings, thereby regulating the biosynthesis of plant coumarins, and flavonoid compounds.

### 4.5 *Bacillus* S40 modulates plant hormones to promote plant growth and increase salt tolerance

Plant hormones are significant signaling molecules between plants and microbes, involved in the regulation of plant growth and development, and they can also be treated as an important factor in mediating plant stress response (Liu et al., 2023). Abscisic acid, indole-3-acetic acid (IAA), cytokinins, jasmonic acid, salicylic acid, and gibberellins were well-known plant growth regulators (Waadt et al., 2022). Research indicates that PGPR can promote plant growth and enhance tolerance to biotic stress by producing phytohormones; they also regulate the homeostasis and signaling of endogenous hormones to support healthy growth under various stress conditions (Ha-Tran et al., 2021). In this study, the three selected strains all produced IAA. Inoculation with strains P2, S37, and S40, the total root length and root fresh weight of *Reaumuria soongorica* seedlings significantly increased compared to the NaCl treatment. Besides, our result showned that inoculation with strain S40 significantly increased the content of metabolites such as 5’-S-Methyl-5′-thioadenosine, jasmonic acid, and jasmonoyl aicd-isoleucine in the leaves of *Reaumuria soongorica* seedlings under NaCl stress (Figure 5E). These metabolites were involved in the plant hormone signal transduction and zeatin biosynthesis pathway. IAA, the first discovered plant hormone, primarily synthesizes via the tryptophan-dependent pathway in plants. PGPRs promote the growth of lateral and adventitious roots through the production of IAA, enhancing nutrient absorption efficiency (He et al., 2023). Strain S40 promoted the growth by the production of IAA and also induced the production of endogenous IAA in *Reaumuria soongorica* seedlings by regulating the biosynthesis of tryptophan. In higher plants, zeatin is the major naturally active cytokinin component that promotes cell division, cell expansion, chloroplast development, and other processes. Inoculation with *Bacillus megaterium* BT22 has been shown to promote Arabidopsis growth by modulating plant hormone signal transduction pathways, including auxins and cytokinins related to cell enlargement and division (Liu et al., 2023). After inoculation with strain S40, the 5’-S-Methyl-5′-thioadenosine content increased in the leaves of *Reaumuria soongorica* seedling compared to the control. This compound was known as one of the main metabolites in zeatin biosynthesis. The findings suggested that strain S40 could protect cells from salt stress damage by modulating cytokinins. Furthermore, Jasmonic acid, ubiquitous in higher plants, is synthesized from the unsaturated fatty acid α-linolenic acid through an enzymatic reaction pathway (Papadopoulou et al., 2023). *Pseudomonas* induced the expression of genes related to jasmonic acid synthesis and α-linolenic acid metabolism in tomato, providing resistance to abiotic stress (Papadopoulou et al., 2023). After inoculation with strain S40, the jasmonic acid and jasmonic acid-isoleucine content increased in the leaves of *Reaumuria soongorica* seedlings. It indicated that Jasmonic acid was an important regulator for the plant’s response to abiotic stress. The results indicate that auxins, zeatin, and jasmonic acid are involved in the stress response of *Reaumuria soongorica* seedlings to stressors, and inoculation with *Bacillus* can alleviate salt stress in plants by modulating plant hormone signal transduction (Lephatsi et al., 2021; Liu et al., 2023).

## 5 Conclusion

The current study identified three strains of PGPR, isolated from the rhizosphere of the desert plant *Reaumuria soongorica*, as salt-tolerant *Bacillaceae* species, which are part of a crucial beneficial microbial population that can be utilized for the reclamation and improvement of saline soils and also for enhancing crop yields in desert regions. Pot experiments demonstrated that under NaCl stress, inoculation with strains P2, S37, and S40 increased the height, total root length, and biomass of *Reaumuria soongorica* seedlings. Strains P2, S37, and S40 promoted the healthy growth of *Reaumuria soongorica* seedlings under salt stress by modulating photosynthetic efficiency and the content of osmoregulatory substances. Comprehensive targeted metabolomic analysis of total metabolites in the leaves of *Reaumuria soongorica* seedlings revealed that inoculation with strain S40 modulated the metabolite content in the leaves, significantly upregulating metabolites involved in plant hormone signal transduction and the phenylalanine biosynthesis pathway. The results suggest that *Bacillaceae* primarily promotes plant growth and salt tolerance by increasing photosynthetic pigments to enhance photosynthetic efficiency, accumulating osmoregulatory substances, regulating hormone signal transduction. The study indicates that PGPR has a positive impact on soil physicochemical properties, nutrient content, and soil enzyme activities. This research focused solely on the effects of salt-tolerant *Bacillus* on plants, with further studies planned to investigate their impact on soil.

## Data availability statement

The datasets presented in this study can be found in online repositories. The names of the repository/repositories and accession number(s) can be found in the article/Supplementary material.

## Author contributions

XB: Conceptualization, Data curation, Investigation, Methodology, Validation, Visualization, Writing – original draft, Writing – review & editing. PC: Conceptualization, Funding acquisition, Project administration, Resources, Supervision, Writing – review & editing. CH: Investigation, Writing – review & editing. XW: Data curation, Investigation, Methodology, Writing – original draft. FZ: Data curation, Investigation, Methodology, Writing – original draft.

## Footnotes

^1^ https://www.ezbiocloud.net/apps

^2^ http://www.genome.jp/kegg/
