# Supplementary material for: Mechanism on the promotion of host growth and enhancement of salt tolerance by Bacillaceae isolated from the rhizosphere of Reaumuria soongorica
Source: Front Microbiol. 2024 May 31;15:1408622. doi: 10.3389/fmicb.2024.1408622 (PMC11176432; doi:10.3389/fmicb.2024.1408622)
Supplement: Supplementary file 1 [file Data_Sheet_1.PDF]

Supplementary Material

Supplementary Tables

Table 1. Statistical analysis results of metabolites in leaves of *Reaumuria soongorica* seedlings inoculated with strain S40.

No.	Compound name	Ion	Rt	Mean CK	Mean S40	Mean S	Mean S40+S	*P*-VALUE	Q-VALUE
1	p-Hydroxyphenylacetylglycine	+	8.17	1.64128E-05	3.79791E-05	1.09119E-05	1.36508E-05	9.06909E-05	0.03627635
2	5'-S-Methyl-5'-thioadenosine	+	3.99	1.79768E-05	0.005953769	0.004810781	0.006403316	0.000202745	0.040549088
3	2-Picolinic acid	+	1.33	0.002078917	0.001594896	0.002496224	0.004250387	0.000347032	0.046270964
4	N-((-)-jasmonoyl)-S-isoleucine	-	10.41	1.68752E-05	1.23393E-05	5.4872E-05	5.22776E-05	0.001509549	0.134477666
5	(±)-Jasmonic acid	-	8.94	0.000265487	0.000323322	0.000517202	0.000573208	0.001830667	0.134477666
6	Abietic acid	+	5.2	0.007004033	0.008158689	0.004057612	0.004189113	0.002184874	0.134477666
7	Sclareol	+	10.62	1.90737E-05	2.01522E-05	1.05964E-05	2.21888E-05	0.002353359	0.134477666
8	N5-(L-1-Carboxyethyl)-L-ornithine	+	2.99	2.14389E-05	3.14178E-05	1.47933E-05	1.60294E-05	0.003727996	0.176010081
9	Pyrrolidonecarboxylic acid	+	0.67	0.002091787	0.008233704	0.002260809	0.003287568	0.003960227	0.176010081
10	7-Ethoxycoumarin	+	10.57	4.41828E-05	3.78037E-05	5.7164E-05	2.47052E-05	0.004895104	0.188342067
11	Chrysin	+	10.94	0.000148145	1.74977E-05	7.24955E-05	6.23035E-05	0.005179407	0.188342067
12	N6-isopentenyladenosine	+	6.1	0.00044023	0.000604859	0.000644929	0.000946845	0.008947701	0.277248593
13	D-Fructose 6-phosphate	-	3.23	0.000125502	0.000114949	5.06942E-05	8.16043E-05	0.009312455	0.277248593
14	N-Feruloyl putrescine	+	4.33	0.000354362	0.000358134	0.000394924	0.000592189	0.010793111	0.277248593
15	Quillaic acid	+	12.94	1.5479E-05	2.99259E-05	4.74664E-05	2.23941E-05	0.010866415	0.277248593
16	Eriodictyol	+	7.9	0.00020035	0.000271803	0.000487836	0.000270294	0.011089944	0.277248593
17	Myricitrin	-	5.97	6.18789E-05	4.28416E-05	0.000154989	4.95313E-05	0.014370243	0.323352198
18	L-Ornithine	+	0.55	0.000342939	0.000419425	0.000977525	0.0010866	0.014550849	0.323352198
19	Calystegine B2	+	2.95	8.68969E-06	1.04272E-05	8.8887E-06	2.13837E-05	0.018419794	0.36000697
20	4-Hydroxycoumarin	-	10.01	3.131E-06	3.28837E-06	2.96972E-06	6.5038E-06	0.018850817	0.36000697
21	Mellein	+	6.45	1.34085E-05	2.59583E-05	1.47274E-05	1.58545E-05	0.02019729	0.36000697
22	2',6'-Dihydroxy 4'-methoxydihydrochalcone	+	10.72	1.36668E-05	2.19204E-05	8.61159E-06	1.34221E-05	0.02103828	0.36000697
23	Gallic acid	-	6.07	4.25894E-05	4.05937E-05	2.19085E-05	3.71679E-05	0.021928976	0.36000697
24	Cauloside A	+	7.4	4.40184E-06	1.14506E-06	4.86118E-06	1.45974E-06	0.022233734	0.36000697
25	Irisflorentin	+	8.64	9.3776E-05	7.82073E-05	4.62322E-05	3.28347E-05	0.023308723	0.36000697
26	1,3,6-Tri-O-galloylglucose	+	5.79	0.000378633	0.000384123	0.000216121	0.000239668	0.023400453	0.36000697
27	Physcion	+	8.24	1.31602E-05	6.56822E-06	4.70428E-06	5.64191E-06	0.02515224	0.372625773
28	1-(4-Hydroxyphenyl)propan-1-one	+	9.9	0.000102169	4.64683E-05	5.61041E-05	4.35277E-05	0.027091226	0.387017517
29	2'-Hydroxygenistein	+	7.59	1.20806E-05	1.67422E-05	3.28747E-05	1.96421E-05	0.033523805	0.462397304
30	4-Isopropylbenzoic acid	+	9	0.000108511	8.14101E-05	6.40269E-05	6.01538E-05	0.036432729	0.468058572

No.	Compound name	Ion	Rt	Mean CK	Mean S40	Mean S	Mean S40+S	*P*-VALUE	Q-VALUE
31	4-Methoxycinnamaldehyde	+	6.8	3.71837E-05	4.28742E-05	2.29892E-05	2.49645E-05	0.036717917	0.468058572
32	L-Phenylalanine;D-(+)-Phenylalanin	+	2.82	0.034211697	0.045900681	0.049091859	0.048253966	0.0385504	0.468058572
33	Epicatechin;(+)-Epicatechin	+	5.08	0.000213322	0.000305891	0.000180495	8.02881E-05	0.038614832	0.468058572
34	N-D-Glucosylarylamine	+	2.69	0.00022197	0.000104612	0.000150671	0.000118153	0.041023159	0.482625403
35	p-Cresol	+	7.28	4.39354E-05	3.8862E-05	3.51015E-05	3.36452E-05	0.04475265	0.511458863
36	Abyssinone V	+	12.6	2.83944E-05	2.6629E-05	3.09344E-05	3.59097E-05	0.049678281	0.52815576
37	Tropine acetate;3-Acetoxytropane	+	3.24	6.02128E-05	8.11415E-05	5.12088E-05	6.92808E-05	0.049760477	0.52815576
38	Sinapyl alcohol	+	4.43	0.000108659	0.000140736	0.00012234	8.91404E-05	0.051011431	0.52815576
39	Methyl cinnamate	+	4.9	0.001211443	0.001341573	0.000750756	0.000646535	0.051676386	0.52815576
40	Cinnamyl cinnamate	+	4.26	0.001631796	0.001820291	0.001627694	0.003016811	0.052815576	0.52815576
41	Naringenin	+	8.86	0.000212487	0.000235924	0.000306055	0.000163315	0.054717558	0.533829837
42	Desoxypeganine	+	8.38	7.8478E-06	6.04775E-06	1.21552E-05	1.22109E-05	0.059753018	0.569076366
43	Geniposidic acid;Geniposidic acid	+	3.7	3.39961E-06	3.297E-06	4.55341E-06	7.06609E-06	0.064681379	0.601687243
44	Rivularine	+	4.35	8.3651E-06	1.08228E-05	5.68245E-06	1.00857E-05	0.067412648	0.602677496
45	Dihydrokavain	+	10.42	1.42693E-05	1.23729E-05	1.59841E-05	7.01283E-06	0.068918089	0.602677496
46	Rutaevin	+	12.58	7.21385E-05	9.61616E-05	5.13943E-05	7.85772E-05	0.069307912	0.602677496
47	Farrerol	+	8.46	1.45693E-05	1.97831E-05	8.33083E-06	1.3429E-05	0.073767627	0.619863869
48	3-Hydroxy-4-methoxycinnamic acid;Isoferulic acid	+	4.87	0.000114984	9.69792E-05	8.02221E-05	3.85274E-05	0.074383664	0.619863869
49	2-Hydroxy-3-(4-hydroxyphenyl)propenoic acid	+	10.05	0.000276822	0.000294015	0.000433656	0.000297347	0.078074534	0.625031426
50	Nordihydroguaiaretic acid	+	6.75	2.98051E-05	8.34597E-06	1.53362E-05	5.92016E-06	0.078128928	0.625031426
51	Bovinic acid	+	6.86	3.0445E-05	3.83884E-05	1.74184E-05	1.81976E-05	0.083169892	0.652312881
52	Curcumol	+	11.14	0.00014988	4.70472E-05	0.000159378	0.000158829	0.086220086	0.659411103
53	Isorhamnetin-3-O-nehesperidine	+	6.02	6.45788E-05	0.000107224	6.59803E-05	7.70419E-05	0.087371971	0.659411103
54	Ergothioneine	+	0.52	0.00023454	0.000238586	0.0002154	0.00021608	0.090511738	0.661305632
55	Guanosine 3',5'-cyclic monophosphate	+	2.26	0.000545683	0.000679595	0.000283445	0.000160763	0.091155712	0.661305632
56	2-Pentylfuran	+	1.11	0.000148495	0.000104507	0.000101877	0.000141763	0.092582788	0.661305632
57	Neolitsine	+	5.04	1.48413E-05	2.92158E-05	1.7645E-05	1.5091E-05	0.095322061	0.663439419
58	alpha-Hexylcinnamaldehyde	+	8.71	2.31084E-05	3.08998E-05	1.57538E-05	3.28544E-05	0.096198716	0.663439419
59	Bruceine D	+	4.84	2.00517E-05	1.37557E-05	2.58819E-05	2.94599E-05	0.100295978	0.668266075
60	Desmethylxanthohumol	+	13.01	0.000165607	0.00026585	0.000235877	0.000256143	0.102976209	0.668266075
61	Methyl gallate	+	4.44	0.000483972	0.00036661	0.00030075	0.000303003	0.105488671	0.668266075
62	Yatein	+	12.6	6.25601E-05	8.90969E-05	9.184E-05	5.74545E-05	0.105562033	0.668266075
63	Salicylic acid	-	7.06	0.000301512	0.00016929	0.00021876	0.000147043	0.105635971	0.668266075
64	Gramine	+	8.73	7.38456E-05	9.35419E-05	0.000117512	9.04091E-05	0.106922572	0.668266075
65	Questinol	+	7.69	1.81235E-05	1.67951E-05	1.04315E-05	4.20165E-06	0.111979367	0.689103795
66	Leukotriene A4	+	5.57	2.10532E-05	2.5913E-05	4.24366E-05	2.23811E-05	0.114431176	0.693522282
67	Pulegone	+	5.9	0.000137353	0.000101801	0.000137553	7.99734E-05	0.119743664	0.697088155
68	Talatisamine	+	4.66	1.54877E-05	1.69325E-05	2.17674E-05	7.60782E-06	0.123042257	0.697088155
69	Loganic acid;Loganic acid	-	7.86	0.000393716	0.000286569	0.000228081	0.000236743	0.123108203	0.697088155

No.	Compound name	Ion	Rt	Mean CK	Mean S40	Mean S	Mean S40+S	*P*-VALUE	Q-VALUE
70	Enol-phenylpyruvate	+	5.08	0.000260643	0.000199932	0.000158761	0.000226215	0.126392693	0.697088155
71	Neoglycyrol	+	13.9	2.19764E-05	4.87227E-05	3.22765E-05	2.61399E-05	0.126780618	0.697088155
72	Syringic acid	+	3.57	0.000141978	0.00010637	0.000111714	8.04764E-05	0.126805331	0.697088155
73	Glutaric acid	-	14.07	0.00011434	0.000164886	0.000202689	0.000130111	0.130648193	0.697088155
74	Dalbergin	+	9.86	4.26898E-06	9.18753E-06	5.81137E-06	5.48975E-06	0.131665735	0.697088155
75	Cianidanol	+	4.5	0.005056178	0.005272763	0.002867344	0.001518725	0.132058314	0.697088155
76	Catalpalactone	+	12.84	5.13168E-05	5.34473E-05	5.31597E-05	3.82588E-05	0.134775974	0.697088155
77	Phlorizin	-	6.41	3.7759E-05	0.000101396	5.86961E-05	3.29457E-05	0.137645372	0.697088155
78	Betulalbuside A	+	4.2	2.93796E-05	1.45548E-05	1.3481E-05	1.19658E-05	0.140724806	0.697088155
79	L-Quebrachitol	+	4.59	4.06059E-05	3.12733E-05	2.48418E-05	1.91754E-05	0.142174517	0.697088155
80	Adenosine 2',3'-cyclic phosphate	+	1.86	4.43308E-05	3.96209E-05	1.51132E-05	1.52913E-05	0.143627366	0.697088155
81	Vanillic acid	+	3.78	5.86376E-05	3.89577E-05	3.79389E-05	3.012E-05	0.143822253	0.697088155
82	2',3,5,7-Tetrahydroxyflavone	+	6.53	0.000727089	0.000946136	0.000469084	0.000760836	0.144407734	0.697088155
83	Tricholomic acid	+	3.61	9.80587E-06	7.45596E-06	6.83408E-06	4.30913E-06	0.145042187	0.697088155
84	beta-Citronellol;(S)-(-)-beta-Citronellol	+	1.41	5.07595E-05	5.5185E-05	2.48186E-05	3.64757E-05	0.146388513	0.697088155
85	Ginsenoside Rg1	+	7.9	4.47625E-06	8.18613E-06	3.33235E-06	4.54579E-06	0.15623988	0.721023628
86	Leukoaminochrome	+	2.42	0.001347334	0.001539438	0.001389998	0.001808837	0.158027751	0.721023628
87	5-Aminovaleric acid	+	1.11	0.003525385	0.004549491	0.004353543	0.00528405	0.158145926	0.721023628
88	Decursin;5-Geranoxy-7-methoxycoumarin	+	13.78	1.10205E-05	1.7285E-05	1.6049E-05	1.68064E-05	0.163910005	0.721023628
89	Tricetin	-	8.05	0.001663793	0.00195914	0.0028365	0.002654496	0.164581944	0.721023628
90	1,5,6-Trihydroxyxanthone	+	10.48	4.79912E-06	2.64697E-06	1.13364E-05	2.84685E-06	0.165033238	0.721023628
91	Fumaric acid	-	0.53	0.000424188	0.000420443	0.000353163	0.000373885	0.166472101	0.721023628
92	Eriobofuran	+	12.52	6.26166E-06	7.23235E-06	4.18582E-06	8.58993E-06	0.168833463	0.721023628
93	3-Hydroxy-3-methylpentane-1,5-dioic acid	-	2.32	9.2432E-05	0.000150035	5.56464E-05	0.000121197	0.172373057	0.721023628
94	Ethyl isovalerate	+	1.19	0.000167738	0.000266896	0.000199595	0.000104661	0.174179663	0.721023628
95	(S)-2-Acetolactate	+	1.32	0.000101806	0.000206899	0.000199196	0.000240591	0.174666106	0.721023628
96	Crotonoside	+	2.4	0.024922959	0.029399549	0.024740882	0.033922649	0.176270356	0.721023628
97	Geranylacetate	+	9.58	0.000117821	9.6142E-05	7.43894E-05	0.000112227	0.176524356	0.721023628
98	Gentioflavin	+	4.93	2.15351E-05	1.42893E-05	2.35208E-05	3.13871E-05	0.176650789	0.721023628
99	12-Hydroxyabietic acid	+	5.53	4.18978E-05	1.77196E-05	3.19209E-05	1.76596E-05	0.181983364	0.735286319
100	Isorhamnetin	+	9.23	0.000387138	0.000281354	0.000483528	0.000513922	0.183996176	0.735984703
101	Kakuol	+	4.58	2.30791E-05	3.20496E-05	3.31011E-05	2.32857E-05	0.189684911	0.745786786
102	Dihydrozeatin	+	4.3	1.09925E-05	6.80499E-06	5.96043E-06	7.84438E-06	0.19017563	0.745786786
103	1-Naphthylacetic acid	+	10.55	2.60061E-05	3.40056E-05	1.62071E-05	2.49243E-05	0.192548888	0.74776267
104	Hydroxysafflor yellow A	+	12.59	0.048344835	0.06898534	0.069445171	0.068614821	0.198270336	0.758296551
105	Ponasterone A	+	6.19	0.00015589	0.000124444	0.000182005	0.000141584	0.201087743	0.758296551
106	(+)-Abscisic acid	-	7.86	0.000123274	0.000145989	0.000136087	0.000102476	0.201627789	0.758296551
107	Cyperotundone	+	11.13	0.000206378	0.000127283	9.33976E-05	0.000123878	0.204519963	0.758296551
108	trans-Zeatin-riboside	+	4.31	5.07897E-05	2.28446E-05	5.58206E-05	5.2702E-05	0.204740069	0.758296551

No.	Compound name	Ion	Rt	Mean CK	Mean S40	Mean S	Mean S40+S	*P*-VALUE	Q-VALUE
109	L-Cysteine	+	4.46	8.00154E-06	4.81216E-06	6.49952E-06	7.03067E-06	0.21425606	0.760530708
110	Genipin	+	4.85	6.55803E-05	3.59282E-05	4.4169E-05	2.63975E-05	0.214940888	0.760530708
111	Asp-Phe methyl ester;Aspartame	+	3.06	0.002147287	0.0032494	0.003868449	0.00553316	0.2154881	0.760530708
112	gamma-Diasarone	+	8.55	0.004168706	0.003427862	0.003105793	0.003575989	0.219317696	0.760530708
113	3-(4-Hydroxyphenyl)-1-propanol	+	3.09	3.07865E-05	2.68905E-05	3.0383E-05	1.72102E-05	0.21951243	0.760530708
114	Dicentrine	+	8.95	1.36116E-05	4.58582E-06	1.81874E-05	1.50907E-05	0.220448394	0.760530708
115	11(R)-HETE	+	5.4	0.000215264	0.00024862	9.03786E-05	0.000129644	0.220541852	0.760530708
116	Isoquercitrin	+	6.06	0.053578813	0.057869668	0.074655968	0.041279608	0.222686725	0.760530708
117	3-Hydroxycoumarin	+	9.94	8.4327E-05	6.28476E-05	4.74991E-05	6.16978E-05	0.224410045	0.760530708
118	(2S,3R,4E)-2-Amino-4-heptadecene-1,3-diol	+	11.14	0.00021058	6.37563E-05	0.000172179	0.000172539	0.228218523	0.760530708
119	L-2-Hydroxyglutaric acid	-	4.12	0.000278795	0.0002425	0.000188994	0.000226944	0.229036626	0.760530708
120	4-Hydroxybenzylamine	+	2.3	7.05371E-06	6.99752E-06	2.88844E-06	9.21272E-06	0.229201195	0.760530708
121	(-)-Maackiain	+	8.48	1.50102E-05	1.39198E-05	1.15014E-05	2.26699E-05	0.230060539	0.760530708
122	L-Tyrosine	+	1.93	0.00104986	0.001284029	0.002042032	0.002260451	0.236630135	0.772760937
123	Altholactone	+	10.56	3.13922E-05	1.70394E-05	3.06802E-05	1.59447E-05	0.239832163	0.772760937
124	Guanidineacetic acid	+	1.11	3.17229E-05	4.14803E-05	3.62309E-05	3.1095E-05	0.243708139	0.772760937
125	Streptomycin	+	12.84	0.000129256	0.000187061	0.000213791	0.000223918	0.245721043	0.772760937
126	Ethyl 3,4,5-trimethoxybenzoate	+	0.53	0.0001352	0.000148578	0.000107885	0.000138411	0.248094579	0.772760937
127	L-Gulose	+	8.71	4.78487E-05	3.67129E-05	2.29249E-05	5.13423E-05	0.253339987	0.772760937
128	3,4-Dihydroxymandelic acid	-	14.2	0.000212536	0.000182736	0.000212139	0.000179239	0.253909047	0.772760937
129	Phenylpyruvic acid	-	5.67	2.06079E-05	2.10245E-05	1.40979E-05	1.33693E-05	0.25447825	0.772760937
130	Erythritol	+	6.17	9.15595E-05	5.63226E-05	8.83671E-05	4.49874E-05	0.255738811	0.772760937
131	Methoxyindoleacetic acid	+	3.76	0.001790667	3.77453E-05	0.003331992	0.003031815	0.256994659	0.772760937
132	Testosterone	+	5.98	1.70687E-05	9.35475E-06	1.16721E-05	1.22395E-05	0.257059657	0.772760937
133	Delta-Nonalactone	+	0.53	0.000694436	0.000916837	0.000641002	0.000781384	0.259177315	0.772760937
134	Anacrotine	+	9.55	6.22135E-06	2.62465E-06	2.79746E-06	2.86059E-06	0.259610517	0.772760937
135	Oleocanthal	+	12.4	0.000116953	0.000132807	0.000101691	4.56795E-05	0.260806816	0.772760937
136	Strychnine	+	3.67	8.88736E-06	7.32502E-06	4.06987E-06	2.53288E-06	0.264717172	0.775957225
137	Dihydroconiferyl alcohol	+	7.99	1.10587E-05	2.20084E-05	1.61293E-05	1.31299E-05	0.265765349	0.775957225
138	4-Sulfobenzoate	+	4.68	7.74167E-05	8.28577E-05	0.000107968	7.10376E-05	0.27278107	0.78934125
139	Mimosine	+	2.37	9.19015E-06	2.79092E-06	2.28861E-06	3.71686E-06	0.274296084	0.78934125
140	3-(2-Hydroxyphenyl)propanoic acid	+	2.84	0.003780923	0.001439288	0.004938563	0.003630293	0.286451451	0.811081726
141	Kaurenoic acid	+	5.9	0.008368763	0.010812003	0.01204914	0.007150306	0.289807887	0.811081726
142	Phenylacetyl-L-glutamine	+	4.71	4.99175E-05	2.84219E-05	3.83445E-05	3.88979E-05	0.290405462	0.811081726
143	3-Octyl alcohol	+	1.23	7.79666E-05	6.37385E-05	9.87167E-05	0.000104139	0.292054553	0.811081726
144	DL-Benzylsuccinic acid	+	4.95	3.84723E-05	8.92876E-05	4.98435E-05	3.94129E-05	0.294034721	0.811081726
145	8-Methylnonenoate	+	2.64	0.000206529	0.000312283	0.000202187	0.000146677	0.296635743	0.811081726
146	(+)-Lyoniresinol 9'-O-glucoside	+	12.8	0.000386889	0.000513416	0.000452365	0.000512041	0.298809418	0.811081726
147	Chelidonic acid	+	1.39	0.000103783	5.10098E-05	8.00862E-05	5.00352E-05	0.299767305	0.811081726

No.	Compound name	Ion	Rt	Mean CK	Mean S40	Mean S	Mean S40+S	*P*-VALUE	Q-VALUE
148	Ganoderenic acid E	+	12.79	6.52079E-05	2.94045E-05	3.61383E-05	2.25121E-05	0.300100238	0.811081726
149	Clovin	+	5.67	4.75424E-06	9.28808E-06	3.18222E-06	7.85194E-06	0.318647618	0.837138834
150	Soyacerebroside I	+	13.8	2.12875E-06	1.14965E-05	2.0044E-05	5.01371E-06	0.31980038	0.837138834
151	Ellagic acid	-	6.12	0.000843466	0.000615653	0.000665531	0.000744131	0.320408134	0.837138834
152	Adenosine	+	2.58	0.018211629	0.008591	0.055861579	0.048568963	0.325914794	0.837138834
153	3,4-Dimethoxycinnamic acid	-	14.08	1.06345E-05	9.20057E-06	7.06137E-06	7.46481E-06	0.326165695	0.837138834
154	Prostaglandin D1	-	10.96	9.83704E-07	5.49807E-06	4.44911E-06	7.53989E-06	0.329549238	0.837138834
155	2-Keto-6-acetamidocaproate	+	1.48	0.000743708	0.000468108	0.000380174	0.000748444	0.329816889	0.837138834
156	2-(4-Hydroxyphenyl)ethanol	-	4.15	0.00010676	8.76059E-05	9.6219E-05	7.59225E-05	0.330913119	0.837138834
157	Clavulanate	-	3.07	8.84354E-06	1.4598E-05	1.04877E-05	1.14394E-05	0.331277282	0.837138834
158	Biochanin A	-	11.38	2.28892E-05	6.62529E-06	7.91337E-06	6.81376E-06	0.332879056	0.837138834
159	Guaiacin	+	7.54	1.3164E-05	1.03094E-05	3.35515E-06	4.72323E-06	0.33551501	0.837138834
160	Xanthosine	+	2.61	0.001853212	0.002220892	0.001888098	0.002347851	0.335994502	0.837138834
161	Micromelin	+	5.13	5.59649E-05	5.49539E-05	0.000100649	5.78618E-05	0.338283095	0.837138834
162	Butin;2'-Hydroxydihydrodaidzein	+	8.83	2.9777E-05	4.63931E-05	5.95744E-05	4.19475E-05	0.339041228	0.837138834
163	Luvangetin	+	4.48	2.57804E-05	1.61068E-05	2.26268E-05	1.61462E-05	0.344450874	0.845278218
164	Deoxylapachol	+	4.93	0.000100689	6.5606E-05	0.000118958	7.39281E-05	0.347335178	0.847158972
165	4-Hydroxy-3-methoxymandelate	+	3.27	2.86968E-05	2.30602E-05	1.77674E-05	2.47771E-05	0.352950299	0.852194538
166	Phosphonoacetate	+	1.88	3.65167E-05	2.85317E-05	1.95984E-05	3.50247E-05	0.354515295	0.852194538
167	Benzaldehyde	+	2.82	0.000769837	0.000757846	0.000911172	0.000971634	0.356153943	0.852194538
168	Isoquinoline;Quinoline	+	6.13	0.005861926	0.004729464	0.004685038	0.00494784	0.357921706	0.852194538
169	5-oxoproline	+	0.56	0.008839369	0.008829394	0.007648702	0.008595482	0.363074261	0.859331001
170	Lauric acid	-	9.74	1.23201E-06	1.9112E-06	1.67739E-06	5.95277E-07	0.366514491	0.859331001
171	Beta-D-Glucose;alpha-D-Glucose	+	3.27	2.48665E-05	1.63723E-05	3.08402E-05	2.16593E-05	0.367364003	0.859331001
172	3-Methyl-2-oxovaleric acid	+	3.8	5.56074E-05	4.69161E-05	3.58327E-05	3.84745E-05	0.371529152	0.864021283
173	Lithospermic acid	+	7.12	5.05143E-06	6.54679E-06	8.09878E-06	8.50705E-06	0.379429283	0.872618897
174	Quercetin 3-O-neohesperidoside	+	6.59	6.69016E-05	7.03217E-05	0.000109464	5.70154E-05	0.385855394	0.872618897
175	6-Hydroxymelatonin	+	5.76	1.56735E-05	1.75589E-05	2.54369E-05	2.13257E-05	0.388149153	0.872618897
176	2,4-Dimethylphenol	+	10.56	4.01703E-05	2.18078E-05	2.58122E-05	2.21719E-05	0.391460389	0.872618897
177	Sciadopitysin	+	12.82	0.000225065	0.000295543	0.000309216	0.000294698	0.391754333	0.872618897
178	Cinnamyl alcohol	+	10.56	2.02183E-05	2.70044E-05	1.81825E-05	1.84263E-05	0.392750768	0.872618897
179	Mulberrofuran Q	+	13.28	0.006762481	0.005007712	0.003198326	0.005545148	0.393402715	0.872618897
180	Eicosadienoic acid	+	10.62	1.77676E-05	1.36405E-05	1.82701E-05	4.3884E-06	0.395303077	0.872618897
181	Jujuboside D	+	12.95	2.35271E-05	2.4589E-05	2.16313E-05	1.23795E-05	0.398572671	0.872618897
182	Guaiacol	+	5.62	2.58331E-05	2.30682E-05	1.36814E-05	2.93575E-05	0.398663913	0.872618897
183	Amygdalin	+	4.88	2.29394E-05	1.28569E-05	5.09705E-06	1.49766E-05	0.400084532	0.872618897
184	Delphinidin	+	6.86	0.000162829	0.000311623	9.1662E-05	6.43088E-05	0.40466956	0.872618897
185	5-Carboxyvanillic acid	+	3.52	1.09019E-05	1.57301E-05	1.69383E-05	2.85223E-05	0.406294594	0.872618897
186	Encecalin	+	10.56	3.79562E-05	6.94818E-05	3.21106E-05	4.32142E-05	0.407722061	0.872618897

No.	Compound name	Ion	Rt	Mean CK	Mean S40	Mean S	Mean S40+S	*P*-VALUE	Q-VALUE
187	Dehydro-Z-notonipetranone	+	13.87	1.26917E-05	1.0488E-05	1.23763E-05	7.2229E-06	0.410717454	0.872618897
188	Piperitenone	+	9.92	9.24553E-06	5.75924E-06	8.79822E-06	7.57206E-06	0.412801491	0.872618897
189	Glutathione oxidized	+	2.52	8.10571E-06	1.06772E-05	6.00298E-06	1.23746E-05	0.416245227	0.872618897
190	Maltotriose	+	1.23	3.004E-06	1.53676E-06	1.93965E-06	2.77209E-06	0.41990632	0.872618897
191	Scoparone	+	5.06	6.58203E-05	8.01778E-05	7.73346E-05	8.556E-05	0.421021761	0.872618897
192	Metanephrine	+	2.13	1.13479E-05	1.6186E-05	1.12118E-05	1.04835E-05	0.421195229	0.872618897
193	Acevaltrate	+	5.56	2.52531E-05	1.07903E-05	3.25626E-05	8.07175E-06	0.424159436	0.872618897
194	2-Hydroxy-3-methylbenzalpyruvate	+	5.07	0.000185745	0.000161468	0.000199932	0.000244866	0.427433984	0.872618897
195	1'-Acetoxychavicol acetate	+	10.55	3.08854E-05	4.92387E-05	3.96437E-05	5.94116E-05	0.428239056	0.872618897
196	Pelargonidin	+	8.98	1.52267E-05	1.19605E-05	2.32481E-05	2.0292E-05	0.42878185	0.872618897
197	Swertiamarin	+	4.82	0.000327705	0.000377215	0.000290734	0.000376447	0.430985564	0.872618897
198	Tabersonine	+	9.78	1.54709E-06	3.61861E-06	4.83174E-06	5.4562E-06	0.439594323	0.872618897
199	Phenylacetylglycine	+	4.92	0.00010861	5.99815E-05	8.69373E-05	0.000103289	0.445415664	0.872618897
200	Napellonine	+	8.48	7.84125E-06	1.08634E-05	1.30201E-05	5.51703E-06	0.446676281	0.872618897
201	Kaempferol-3-O-rutinoside	+	6.29	0.006846282	0.00947719	0.006910256	0.007747022	0.448048242	0.872618897
202	Cinnamyl acetat	+	10.56	3.51193E-05	4.77141E-05	2.39117E-05	2.33216E-05	0.449008472	0.872618897
203	7-Methylguanine	+	1.56	1.67009E-05	2.29744E-05	1.72479E-05	2.48592E-05	0.449632651	0.872618897
204	Curcolone	+	8.37	1.19039E-05	1.27207E-05	1.19271E-05	2.01042E-05	0.45330299	0.872618897
205	Baicalin	+	7.26	5.17543E-05	3.43541E-05	3.59914E-05	3.48588E-05	0.454593051	0.872618897
206	Myristoleic acid	+	4.96	4.93764E-05	6.45519E-05	6.65916E-05	6.43818E-05	0.45636714	0.872618897
207	Tripalmitin	+	12.58	3.19014E-05	1.89425E-05	2.26677E-05	1.88378E-05	0.457164602	0.872618897
208	Phenyl acetate	+	10.56	0.000128117	0.000143076	8.13728E-05	0.000123923	0.458194131	0.872618897
209	Jangomolide	+	13.79	3.48141E-05	5.9959E-05	3.33388E-05	3.68143E-05	0.458998053	0.872618897
210	Undecanolactone	+	0.57	0.000390792	0.000415593	0.000333912	0.000374695	0.460348304	0.872618897
211	Homoeriodictyol	+	6.83	0.000122946	0.000102183	0.00012126	6.92491E-05	0.461071826	0.872618897
212	Neocnidilide	+	6.81	6.61506E-05	9.49423E-05	9.73863E-05	6.27549E-05	0.462488016	0.872618897
213	Anthranilic acid	+	2.88	4.37541E-05	5.00205E-05	3.89724E-05	3.0253E-05	0.47553489	0.893023267
214	ent-16beta,17-dihydroxy-9(11)-kauren-19-oic acid	+	7.15	7.18415E-06	2.98678E-06	6.63236E-06	4.9988E-06	0.480865161	0.89480669
215	Lusianthridin	+	9.99	8.91531E-06	7.25328E-06	1.18989E-05	1.68355E-05	0.481177311	0.89480669
216	Fucoxanthin	+	12.49	1.73587E-05	2.82874E-05	1.22148E-05	3.34002E-05	0.483514456	0.89480669
217	2,5-Dihydroxybenzaldehyde	+	5.09	0.000351519	0.000187912	0.000302038	0.00016352	0.485432629	0.89480669
218	Cochlearine	+	13.76	3.34376E-05	2.76371E-05	3.92283E-05	4.59918E-05	0.490003763	0.896802347
219	Quercetin	-	8.07	0.000135251	0.000143378	0.000197056	0.000183693	0.493188863	0.896802347
220	N'-Formylkynurenine	+	9.51	1.39279E-05	1.79948E-05	1.1365E-05	1.55227E-05	0.495410154	0.896802347
221	Gamabufotalin;Telocinobufagin	+	6.8	1.42061E-05	1.02231E-05	1.06326E-05	1.74357E-05	0.496790738	0.896802347
222	Lonicerin	+	6.08	5.74897E-06	6.8948E-06	1.40654E-05	6.03581E-06	0.497907802	0.896802347
223	N-Acetyl-L-glutamic acid	+	3.75	1.68839E-05	2.03317E-05	2.50155E-05	2.51368E-05	0.499967309	0.896802347
224	Xanthohumol	-	10.96	1.89093E-05	9.71682E-06	1.32057E-05	1.50259E-05	0.502773245	0.897809365
225	Licoisoflavone A	+	10.98	7.11521E-06	3.57074E-06	3.18308E-06	2.53523E-06	0.505070348	0.897902841

No.	Compound name	Ion	Rt	Mean CK	Mean S40	Mean S	Mean S40+S	*P*-VALUE	Q-VALUE
226	Albiflorin	-	6.23	3.61652E-05	0.000129047	2.17534E-05	0.000118717	0.507556498	0.898330084
227	CYS-GLY;Cysteinylglycine	+	1.38	6.82887E-05	0.000190777	1.62203E-05	6.42459E-06	0.527450323	0.915229065
228	Fisetin	+	7.03	3.95656E-05	2.12433E-05	4.24819E-05	3.23379E-05	0.530316863	0.915229065
229	gamma-Fagarine	+	9.3	3.04576E-06	3.46754E-06	1.8915E-06	1.63781E-06	0.531341816	0.915229065
230	Lactupicrin	+	13.8	1.00937E-05	1.16637E-05	8.22434E-06	7.35781E-06	0.531472655	0.915229065
231	5-Tricosyl-1,3-benzenediol	+	13.85	5.52826E-05	6.37004E-05	5.44103E-05	7.71735E-05	0.53364692	0.915229065
232	Protocatechuic acid;protocatechuic acid	-	3.43	0.000379917	0.000349488	0.000430864	0.000423359	0.534262138	0.915229065
233	3-Hydroxy-2-methylpyridine	+	1.44	1.11127E-05	9.63205E-06	7.80362E-06	9.00738E-06	0.534873583	0.915229065
234	Hydrocinnamic acid	+	11.25	4.48445E-05	1.75431E-05	6.85868E-05	4.82041E-05	0.536301793	0.915229065
235	Bufotenine	+	10.57	6.10034E-06	3.85996E-06	5.42804E-06	1.16095E-05	0.538791426	0.915229065
236	2(3H)-Benzothiazolethione	+	2.57	3.40827E-05	2.3729E-05	1.71986E-05	2.06115E-05	0.539985148	0.915229065
237	Avicularin	-	6.47	0.000110804	5.06658E-05	0.000144656	0.000115768	0.542870665	0.916237409
238	Citronellyl acetate	+	10.48	7.71372E-05	4.29235E-05	5.54606E-05	7.79401E-05	0.553015734	0.928301467
239	Rotenone	+	12.92	4.01635E-05	6.55464E-05	7.42192E-05	6.83865E-05	0.554660127	0.928301467
240	Chalepensin	+	7.62	4.03866E-05	3.17355E-05	3.07784E-05	2.97698E-05	0.56069667	0.928301985
241	Lucidadiol	+	12.64	1.41018E-05	1.80326E-05	1.94229E-05	1.09841E-05	0.562330257	0.928301985
242	Glucosamine	+	2.6	1.45071E-05	1.76864E-05	2.24577E-05	1.77384E-05	0.573889821	0.928301985
243	1,7-Diphenyl-4-hepten-3-one	+	5.9	2.0004E-05	2.91328E-05	4.16039E-05	2.17826E-05	0.576209003	0.928301985
244	Gallocatechin	+	3.36	0.004491135	0.004801976	0.003936783	0.000904442	0.576418697	0.928301985
245	Benzyl acetate	+	10.86	5.72797E-05	6.52372E-05	8.04005E-05	4.37569E-05	0.578594537	0.928301985
246	22-Dehydroclerosterol	+	12.59	3.59181E-05	2.9757E-05	4.87448E-05	3.55141E-05	0.582030301	0.928301985
247	Deoxyguanosine	-	2.72	3.16561E-06	2.90651E-05	4.98995E-05	3.20282E-05	0.584748472	0.928301985
248	Catechin	-	6.05	2.0504E-05	2.40776E-05	2.30356E-05	1.16062E-05	0.589550317	0.928301985
249	Denudatine	+	1.59	0.000228287	0.000261054	0.000149272	0.000221995	0.59207209	0.928301985
250	Swertiajaponin	+	13.86	0.00031315	0.000202044	0.000275827	0.000233347	0.59472113	0.928301985
251	N1-Methyl-2-pyridone-5-carboxamide	+	2.63	8.5563E-05	7.96001E-05	8.51068E-05	0.00011144	0.59543659	0.928301985
252	Sugiol	+	8.21	8.07831E-05	4.69534E-05	3.38623E-05	0.000101284	0.601221077	0.928301985
253	Capsanthin	+	12.8	5.44555E-05	7.35E-05	7.43222E-05	7.34008E-05	0.601799845	0.928301985
254	Purine	+	2.05	0.00404622	0.002683717	0.012905582	0.0025545	0.602266733	0.928301985
255	5-Phenyl-1,3-oxazinane-2,4-dione	+	2.8	1.27175E-05	2.06309E-05	2.46886E-05	1.35777E-05	0.605577473	0.928301985
256	Ganoderic acid A, B, C;	+	12.39	4.47025E-05	6.40551E-05	8.14574E-05	4.95577E-05	0.606747612	0.928301985
257	DG(16:0/16:0/0:0)	+	13.42	0.068578399	0.113485068	0.117437892	0.09140444	0.606841925	0.928301985
258	(-)-Epicatechin gallate	-	6.05	0.00117081	0.001281639	0.001168291	0.000568434	0.609005615	0.928301985
259	Carnosic acid	-	8.49	0.00143668	0.001244045	0.001170204	0.00121121	0.610102566	0.928301985
260	Bergaptol	+	7.44	1.66668E-05	1.69866E-05	1.3942E-05	1.08308E-05	0.621687373	0.928301985
261	5,6-DHET	+	13.03	5.94789E-05	7.16959E-05	7.18629E-05	9.14067E-05	0.62183934	0.928301985
262	10-Gingerol	+	12.44	5.18249E-05	5.67038E-05	7.37972E-05	4.47898E-05	0.623055096	0.928301985
263	Tryptamine	+	2.86	8.77176E-06	9.43945E-06	6.79549E-06	1.03828E-05	0.623737301	0.928301985
264	Methylisopelletierine	+	4.11	2.1454E-05	2.01212E-05	1.19616E-05	2.28959E-05	0.627787342	0.928301985

No.	Compound name	Ion	Rt	Mean CK	Mean S40	Mean S	Mean S40+S	*P*-VALUE	Q-VALUE
265	Ethyl gallate	+	8.86	2.08015E-05	2.58545E-05	3.683E-05	2.72197E-05	0.630862712	0.928301985
266	Cevadine	+	13.28	0.004967397	0.007114094	0.008394409	0.005571824	0.631417128	0.928301985
267	Isokadsuranin	+	12.55	6.15426E-06	7.36243E-06	1.06293E-05	6.67979E-06	0.633743233	0.928301985
268	Karacoline	+	5.21	1.21795E-05	9.62323E-06	4.28064E-06	1.19281E-05	0.634304914	0.928301985
269	Convallatoxin	+	12.81	9.99457E-06	6.95119E-06	6.2016E-06	5.51309E-06	0.634737595	0.928301985
270	N-Methylcytisine	+	1.14	1.59818E-05	1.13428E-05	1.4816E-05	1.63029E-05	0.634914026	0.928301985
271	5-(3-Pyridyl)-2-hydroxytetrahydrofuran	+	2.8	2.89753E-05	1.13848E-05	1.98766E-05	2.6414E-05	0.635492471	0.928301985
272	Enoxacin	+	10.84	7.61415E-05	7.47678E-05	7.0595E-05	4.99227E-05	0.639051467	0.928301985
273	Pimelic acid	+	0.55	0.008221685	0.008478777	0.008458255	0.007888539	0.647736979	0.928301985
274	cis-Gondoic acid	+	13.4	9.12307E-05	9.92464E-05	0.000109381	0.000143883	0.650957581	0.928301985
275	trans-Hinokiresinol	+	10.62	1.28577E-05	2.02126E-05	2.06748E-05	1.32321E-05	0.654107052	0.928301985
276	Acetyl tryptophan	+	7.97	9.34543E-06	7.71276E-06	1.11302E-05	8.92208E-06	0.657137602	0.928301985
277	Kynurenic acid	+	4.36	0.002839771	0.00239724	0.00158717	0.00238544	0.657254264	0.928301985
278	Alloimperatorin	+	8.83	2.33738E-05	2.40783E-05	3.22183E-05	2.00158E-05	0.658118113	0.928301985
279	N1-Methyl-4-pyridone-3-carboxamide	+	2.66	0.000496716	0.000422078	0.000437807	0.000405787	0.659254761	0.928301985
280	5'-Deoxyadenosine	+	2.41	0.001724479	0.001794838	0.002274915	0.001604405	0.660078357	0.928301985
281	1H-Indole-2,3-dione	+	2.79	5.08259E-05	4.08886E-05	3.59357E-05	3.10834E-05	0.660538448	0.928301985
282	3,4-Dihydroxyphenylglycol	+	3.08	0.002035075	0.002713708	0.002206474	0.001620189	0.661614656	0.928301985
283	Angelicin	+	8.97	2.90368E-05	3.01898E-05	2.22283E-05	2.75248E-05	0.666790452	0.928301985
284	Cephalotaxine	+	4.93	1.40037E-05	2.35484E-05	1.43316E-05	2.0324E-05	0.667695953	0.928301985
285	6-Aminocaproic acid	+	1.84	0.001482559	0.003178553	0.002323801	0.002537228	0.668284012	0.928301985
286	Lincomycin	+	12.44	0.000309682	0.000359142	0.000308957	0.000261293	0.670092691	0.928301985
287	Toluene-cis-dihydrodiol	+	1.78	1.69769E-05	2.34295E-05	1.87729E-05	1.30434E-05	0.672894664	0.928301985
288	Limonexic acid	+	12.55	0.000622267	0.000475217	0.000747012	0.00059728	0.673602738	0.928301985
289	1-(beta-D-Ribofuranosyl)-1,4-dihydronicotinamide	+	2.8	3.84881E-06	4.96954E-06	7.62239E-06	7.70929E-06	0.673731551	0.928301985
290	Caffeic acid	+	4.44	3.28362E-05	4.50722E-05	4.32124E-05	3.18688E-05	0.675724566	0.928301985
291	Peonidin-3-glucoside	+	6.93	0.001405213	0.001232713	0.001340802	0.001217463	0.677087657	0.928301985
292	5-Hydroxyindole-3-acetic acid	+	10.55	5.39869E-06	7.62346E-06	7.55342E-06	1.20765E-05	0.677660449	0.928301985
293	(-)-Carvone	+	10.86	1.27298E-05	1.74956E-05	1.17443E-05	1.48876E-05	0.681353878	0.930175943
294	Rutin	+	5.85	0.235144283	0.277153429	0.300722974	0.227028875	0.693126341	0.938983147
295	Moracin C	+	13.99	0.001705618	0.00200118	0.001361087	0.001391709	0.694337032	0.938983147
296	1,2,5,6-Tetrahydro-4H-pyrrolo[3,2,1-ij]quinolin-4-one	+	10.56	3.44375E-05	2.30003E-05	2.37784E-05	2.31802E-05	0.69662373	0.938983147
297	Isoxanthopterin	+	3.26	6.03073E-05	8.67756E-05	7.78887E-05	6.75535E-05	0.697194987	0.938983147
298	Ethyl cinnamate	+	4.85	0.000170792	0.000153437	0.0001229	0.000148009	0.701964895	0.942234759
299	Tussilagine	+	3.85	1.07348E-05	8.41868E-06	6.07159E-06	9.67559E-06	0.704934512	0.942751462
300	Canthaxanthin	+	12.82	0.00040077	0.000451402	0.000407005	0.000523608	0.710550001	0.942751462
301	1H-Indole-3-carboxylic acid;Indole-3-carboxylic	-	0.53	6.51828E-05	6.78809E-05	6.24678E-05	5.82333E-05	0.711913723	0.942751462
302	3,4-Dihydroxyphenylacetaldehyde	+	4.05	1.68534E-05	1.27411E-05	1.79081E-05	1.85094E-05	0.714652528	0.942751462
303	Terpinine-4-ol	+	5.45	0.000133085	9.70664E-05	0.00013381	0.000126703	0.716830831	0.942751462

No.	Compound name	Ion	Rt	Mean CK	Mean S40	Mean S	Mean S40+S	*P*-VALUE	Q-VALUE
304	N-γ-Acetyl-N-2-Formyl-5-methoxykynurenamine	+	3.79	1.58828E-05	1.12584E-05	1.56322E-05	1.51429E-05	0.718988547	0.942751462
305	Lutein	+	13.14	2.95562E-05	3.50045E-05	5.58894E-05	3.94742E-05	0.718999403	0.942751462
306	Furan-3-carboxylic acid	-	13.77	2.13782E-06	2.9036E-06	2.753E-06	2.80531E-06	0.722276034	0.942751462
307	N-Methyltyramine	+	2.38	5.54596E-06	3.83912E-06	5.90191E-06	3.38897E-06	0.723561747	0.942751462
308	Riboflavine	+	5.06	0.002432569	0.002527546	0.002395422	0.002784666	0.728918802	0.946647795
309	D-Alanyl-D-Alanine	+	4.33	7.5011E-06	5.434E-06	5.51517E-06	4.80974E-06	0.734196073	0.947872313
310	Cheilanthifoline	+	14.13	4.15657E-06	4.28539E-06	4.33859E-06	2.37514E-06	0.737005395	0.947872313
311	Ascorbic acid	+	1.13	0.000178569	6.50114E-05	0.000365462	6.27227E-05	0.7428206	0.947872313
312	3-Aminoisobutanoic acid	+	0.94	9.09543E-05	3.56834E-06	5.20873E-06	7.15813E-06	0.745036577	0.947872313
313	Palmitoylethanolamide	+	12.61	0.000343178	0.000483345	0.000462541	0.000493483	0.745395095	0.947872313
314	3',4',7-Trihydroxyisoflavone	+	8.98	2.15275E-05	1.56435E-05	1.42437E-05	1.80327E-05	0.7492264	0.947872313
315	Pogostone	+	4.95	2.82814E-05	3.53706E-05	3.27752E-05	2.58007E-05	0.750389965	0.947872313
316	Trans-caffeic acid	+	3.31	2.47411E-05	2.36252E-05	2.50978E-05	1.88079E-05	0.750805305	0.947872313
317	2,6-Dimethyl-7-octene-2,3,6-triol	+	3.82	6.47644E-06	8.77372E-06	8.48761E-06	8.73757E-06	0.751847548	0.947872313
318	Puerarin	+	4.89	0.002040417	0.001393472	0.00198388	0.00202025	0.753935497	0.947872313
319	Cleomiscosin A	+	5.74	0.002061901	0.001703487	0.001483682	0.001536884	0.758039099	0.947872313
320	Hesperetin 7-O-glucoside	+	6.07	8.65124E-05	9.29318E-05	0.000110274	9.44868E-05	0.759337885	0.947872313
321	Aristolindiquinone	+	11.14	0.000161839	9.90386E-05	0.000160118	0.000251207	0.763373937	0.947872313
322	Cineole	+	6.78	7.99771E-05	9.57424E-05	0.000104512	0.000101494	0.764351212	0.947872313
323	Taxifolin	+	6.28	0.000466359	0.000557824	0.000546756	0.000464718	0.765553851	0.947872313
324	Corydaline	+	12.94	4.46002E-06	4.75357E-06	2.97953E-06	3.9052E-06	0.77071609	0.947872313
325	Phloretin	+	8.85	4.03503E-05	5.72131E-05	3.33285E-05	5.2854E-05	0.77164922	0.947872313
326	Isocitrate	-	3.72	3.40384E-05	4.3042E-05	4.68025E-05	4.44518E-05	0.773029377	0.947872313
327	N-p-Coumaroyl putrescine	+	4.16	1.89899E-05	1.3791E-05	9.39616E-06	1.62656E-05	0.774885616	0.947872313
328	5-Hydroxylysine	+	0.55	2.89065E-05	4.04506E-05	3.13066E-05	3.41994E-05	0.781415558	0.949993918
329	(-)-Epigallocatechin gallate	-	5.14	0.000625402	0.000648119	0.000659992	0.000358933	0.782275143	0.949993918
330	Methyl hexadecanoic acid	+	9.78	0.000123126	0.000126058	0.000111568	0.000134316	0.784773108	0.949993918
331	Podophyllotoxin	+	9.66	4.13377E-06	2.17915E-06	3.62325E-06	4.15595E-06	0.786119967	0.949993918
332	Oxoadipic acid	-	1.65	6.74614E-06	6.0456E-06	5.25773E-06	5.95891E-06	0.789629201	0.950410359
333	Mitraphylline	+	13.02	0.000193169	0.000174201	0.000187186	0.000213589	0.793359082	0.950410359
334	Oleamide	+	12.54	0.000300172	0.000327675	0.000392437	0.000295576	0.795383463	0.950410359
335	6-(Furfurylamino)purine;Kinetin	+	4.55	9.9783E-06	9.66376E-06	7.83716E-06	6.75633E-06	0.795968675	0.950410359
336	Ganoderic acid F	+	12.97	0.000274849	0.000259746	0.000218005	0.000209225	0.799661616	0.951978114
337	D-Arabinose 5-phosphate	+	6.29	4.45818E-05	3.005E-05	3.5396E-05	3.55276E-05	0.802888182	0.952983005
338	(R)-Acetoin	+	0.53	0.000735028	0.000710539	0.000625312	0.00066595	0.807426132	0.953857348
339	Sitostenone	+	13.71	6.89314E-05	7.86177E-05	8.05511E-05	9.13938E-05	0.814212531	0.953857348
340	L-Hypoglycin A	+	0.52	0.00012644	0.000135453	0.000145668	0.000107227	0.819171032	0.953857348
341	Hordenine	+	3.04	1.12374E-05	2.27918E-05	1.52143E-05	2.07769E-05	0.822750063	0.953857348
342	Vomicine	+	5.37	7.38804E-06	1.21846E-05	7.23952E-06	8.79113E-06	0.825820601	0.953857348

No.	Compound name	Ion	Rt	Mean CK	Mean S40	Mean S	Mean S40+S	*P*-VALUE	Q-VALUE
343	2-Aminomuconate semialdehyde	+	1.19	5.09876E-05	5.27347E-05	5.50638E-05	4.43443E-05	0.828270681	0.953857348
344	Evoxine	+	6.49	1.51307E-05	9.05949E-06	8.39202E-06	9.45994E-06	0.831373871	0.953857348
345	Isoliquiritin; Isoliquiritoside	+	6.94	1.70482E-05	1.31594E-05	1.27027E-05	1.71013E-05	0.831464428	0.953857348
346	Mannose 6-phosphate	+	13.03	0.000558175	0.000455992	0.000536074	0.000473088	0.831485989	0.953857348
347	Perillyl aldehyde	+	10.86	0.00015213	0.000132124	0.000169102	0.000137332	0.831511937	0.953857348
348	Homopterocarpin	+	9.07	1.26522E-05	1.49749E-05	1.53571E-05	1.95608E-05	0.834206514	0.953857348
349	Phenethyl alcohol;2-Phenylethanol	+	3.37	0.067736804	0.046234491	0.081256498	0.046210362	0.835451397	0.953857348
350	Ligustilide	+	2.8	2.74213E-05	2.22271E-05	2.07775E-05	2.91129E-05	0.839939788	0.953857348
351	Styrene	+	3.37	0.018970888	0.013751578	0.023466567	0.01314268	0.840462212	0.953857348
352	L-Valine	+	0.93	0.004579284	0.003977801	0.004728694	0.004656395	0.842121458	0.953857348
353	Trachelogenin	+	5	1.26089E-05	1.66272E-05	1.2556E-05	1.28582E-05	0.842826959	0.953857348
354	2,4-dienoate	+	8.73	2.379E-05	2.5038E-05	2.02196E-05	2.65875E-05	0.846922779	0.953857348
355	4-Hydroxyphenylacetylglutamic acid	+	12.99	0.144816349	0.115855504	0.127822179	0.127942883	0.851777088	0.953857348
356	Dopamine	-	6.77	8.96885E-06	9.91778E-06	8.33158E-06	7.17293E-06	0.852051401	0.953857348
357	Malvidin-3-O-galactoside	+	7.75	4.42724E-05	2.90833E-05	3.52036E-05	3.16104E-05	0.853054483	0.953857348
358	Ferulic acid; Trans-Ferulic acid	+	4.86	0.000254395	0.00026093	0.000262605	0.000219999	0.856293465	0.953857348
359	Terpinolene	+	10.57	0.000168074	0.000191872	0.000177324	0.000139949	0.856687275	0.953857348
360	(-)-Fenchone	+	3.07	0.007523552	0.0091433	0.00838223	0.007043358	0.860815434	0.953857348
361	Shionone	+	12	9.41318E-05	8.2808E-05	7.89511E-05	9.52062E-05	0.860856257	0.953857348
362	Kaempferol	+	9.39	4.1726E-06	5.27917E-06	3.90274E-06	4.57461E-06	0.86823331	0.957656301
363	2'-Deoxyuridine;Deoxyuridine	+	2.67	4.28413E-05	3.8258E-05	2.85874E-05	4.23296E-05	0.869073093	0.957656301
364	2-Hydroxyxanthone	+	7.27	9.80926E-06	1.24161E-05	1.18925E-05	1.10303E-05	0.877991218	0.964163599
365	8-Azabicyclo-3.2.1-octan-3-ol	+	14.09	9.88327E-06	6.9942E-06	1.05449E-05	8.80529E-06	0.879799284	0.964163599
366	Quercitrin	-	6.59	0.000460735	0.000524755	0.000566519	0.000516598	0.88412695	0.966258962
367	Cyanidin-3-O-rutinoside chloride	+	13.55	2.79561E-05	2.44126E-05	2.17211E-05	2.10458E-05	0.897695836	0.974373836
368	Melatonin	+	10.42	7.08357E-06	1.11135E-05	1.06054E-05	9.10789E-06	0.898748699	0.974373836
369	Dictamnine	+	9.9	5.56091E-05	4.64598E-05	4.67801E-05	5.20888E-05	0.898859863	0.974373836
370	Niazirin	+	12.54	1.35434E-05	1.2221E-05	1.22591E-05	1.76011E-05	0.903276112	0.976514715
371	7-(4-Hydroxyphenyl)-1-phenyl-4-hepten-3-one	+	12.53	0.001501329	0.001361153	0.001643925	0.001348666	0.920851942	0.990990981
372	Deethylatrazine	+	3.81	0.00180631	0.002457234	0.001601269	0.001689774	0.921621612	0.990990981
373	Oleic acid;Vaccenic acid;Petroselinic acid	+	13.03	0.031183999	0.031609586	0.036552095	0.032165491	0.930108731	0.991548155
374	2,3-Dehydrosilybin A	+	5.53	9.20797E-06	7.80857E-06	1.02116E-05	1.20828E-05	0.930484684	0.991548155
375	Spinosin	-	5.79	0.000355487	0.000344987	0.000497909	0.000437027	0.936594093	0.991548155
376	Glucose 1-phosphate	+	13	0.00784532	0.008519881	0.008509274	0.007766082	0.939185143	0.991548155
377	Artemisinin	+	13.02	0.007374612	0.006494457	0.007196657	0.007376564	0.944281178	0.991548155
378	Sinalbin	+	13.23	2.48283E-05	2.92636E-05	3.02253E-05	2.66656E-05	0.944940219	0.991548155
379	Miltirone	+	12.98	0.043863925	0.039090892	0.043803053	0.041188021	0.948130933	0.991548155
380	Octadecanamide	+	13.02	0.001872662	0.001723717	0.001758886	0.001617306	0.949057838	0.991548155
381	Coniferyl alcohol	+	3.27	2.31689E-05	2.04012E-05	2.19681E-05	2.00514E-05	0.956066268	0.991548155

No.	Compound name	Ion	Rt	Mean CK	Mean S40	Mean S	Mean S40+S	*P*-VALUE	Q-VALUE
382	Vanillin	+	5.9	0.000455034	0.000486502	0.000421437	0.000535764	0.95640117	0.991548155
383	Palmitic acid	+	12.92	0.003565937	0.003560857	0.003535442	0.003056013	0.956703585	0.991548155
384	Narcissoside	-	6.38	0.001798741	0.001633237	0.001816396	0.001538828	0.957549772	0.991548155
385	9-Methoxy-alpha-lapachone	+	6.51	2.7539E-05	2.41612E-05	2.38449E-05	2.67141E-05	0.96039247	0.991548155
386	Sipeimine	+	13.04	2.39716E-05	2.85792E-05	2.7988E-05	2.72194E-05	0.961472911	0.991548155
387	3-Indolebutyric acid	+	9	5.9983E-05	6.55252E-05	6.58162E-05	6.21189E-05	0.963055941	0.991548155
388	Amabiline;Supinine	+	12.99	0.0012912	0.001209688	0.00119535	0.00111653	0.964839394	0.991548155
389	Putrescine	+	0.24	0.000414611	0.000399569	0.000401172	0.00040474	0.967698002	0.991548155
390	Bergamotine	+	13.03	0.000365745	0.000318132	0.000328222	0.000345353	0.968436857	0.991548155
391	Wedelolactone	-	10.22	8.0374E-06	6.64239E-06	7.55837E-06	8.85885E-06	0.969238322	0.991548155
392	8,9-DiHETrE	+	13.03	0.003662744	0.003505051	0.00339162	0.003362894	0.976205859	0.993355868
393	2-Hydroxycinnamic acid	+	5.44	0.000185086	0.000182831	0.00013584	0.000161152	0.977062881	0.993355868
394	Vasicinol	+	10.57	1.21412E-05	1.26265E-05	1.4304E-05	1.2616E-05	0.983633326	0.993355868
395	Panaxynol	+	12.57	0.00023921	0.000217114	0.000222128	0.000238439	0.984566771	0.993355868
396	Stearic Acid	+	13.02	0.000297751	0.000313055	0.000301773	0.000313339	0.98465348	0.993355868
397	2-Carboxybenzaldehyde	+	3.07	5.05061E-05	5.46812E-05	4.89021E-05	5.08614E-05	0.985905699	0.993355868
398	Angustifoline	+	10.56	1.03554E-05	8.39891E-06	1.06677E-05	9.41096E-06	0.989831476	0.994805503
399	Glycerophosphocholine	+	12.87	0.000669062	0.000645613	0.000630733	0.00061922	0.99622073	0.998717524
400	Octyl Gallate	+	12.97	0.000796435	0.000786534	0.000792732	0.000775078	0.999274176	0.999274176

Note: Ion: Positive and negative ion modes of mass spectrometry; Rt: Chromatographic retention time of this substance; Mean CK, Mean S, Mean S40, and Mean S40+S: The relative quantitative mean value of different groups; *P*-VALUE: *P* value of the variance of the substance in the group comparison; Q-VALUE: The result of hypothesis test statistics (*P* value) corrected by multiple hypothesis tests.

Table 2. Statistical analysis results of Differential metabolites in leaves of *Reaumuria soongorica* seedlings inoculated with strain S40.

No.	Compound name	Ion	Rt	Mean CK	Mean S40	Mean S	Mean S40+S	*P*-VALUE	Q-VALUE
1	p-Hydroxyphenylacetylglycine	+	8.17	1.64128E-05	3.79791E-05	1.09119E-05	1.36508E-05	9.06909E-05	0.03627635
2	5'-S-Methyl-5'-thioadenosine	+	3.99	1.79768E-05	0.005953769	0.004810781	0.006403316	0.000202745	0.040549088
3	2-Picolinic acid	+	1.33	0.002078917	0.001594896	0.002496224	0.004250387	0.000347032	0.046270964
4	N-((-)-jasmonoyl)-S-isoleucine	-	10.41	1.68752E-05	1.23393E-05	5.4872E-05	5.22776E-05	0.001509549	0.134477666
5	(±)-Jasmonic acid	-	8.94	0.000265487	0.000323322	0.000517202	0.000573208	0.001830667	0.134477666
6	Abietic acid	+	5.2	0.007004033	0.008158689	0.004057612	0.004189113	0.002184874	0.134477666
7	Sclareol	+	10.62	1.90737E-05	2.01522E-05	1.05964E-05	2.21888E-05	0.002353359	0.134477666
8	N5-(L-1-Carboxyethyl)-L-ornithine	+	2.99	2.14389E-05	3.14178E-05	1.47933E-05	1.60294E-05	0.003727996	0.176010081
9	Pyrrolidonecarboxylic acid	+	0.67	0.002091787	0.008233704	0.002260809	0.003287568	0.003960227	0.176010081
10	7-Ethoxycoumarin	+	10.57	4.41828E-05	3.78037E-05	5.7164E-05	2.47052E-05	0.004895104	0.188342067
11	Chrysin	+	10.94	0.000148145	1.74977E-05	7.24955E-05	6.23035E-05	0.005179407	0.188342067
12	N6-isopentenyladenosine	+	6.1	0.00044023	0.000604859	0.000644929	0.000946845	0.008947701	0.277248593
13	D-Fructose 6-phosphate	-	3.23	0.000125502	0.000114949	5.06942E-05	8.16043E-05	0.009312455	0.277248593

No.	Compound name	Ion	Rt	Mean CK	Mean S40	Mean S	Mean S40+S	*P*-VALUE	Q-VALUE
14	N-Feruloyl putrescine	+	4.33	0.000354362	0.000358134	0.000394924	0.000592189	0.010793111	0.277248593
15	Quillaic acid	+	12.94	1.5479E-05	2.99259E-05	4.74664E-05	2.23941E-05	0.010866415	0.277248593
16	Eriodictyol	+	7.9	0.00020035	0.000271803	0.000487836	0.000270294	0.011089944	0.277248593
17	Myricitrin	-	5.97	6.18789E-05	4.28416E-05	0.000154989	4.95313E-05	0.014370243	0.323352198
18	L-Ornithine	+	0.55	0.000342939	0.000419425	0.000977525	0.0010866	0.014550849	0.323352198
19	Calystegine B2	+	2.95	8.68969E-06	1.04272E-05	8.8887E-06	2.13837E-05	0.018419794	0.36000697
20	4-Hydroxycoumarin	-	10.01	3.131E-06	3.28837E-06	2.96972E-06	6.5038E-06	0.018850817	0.36000697
21	Mellein	+	6.45	1.34085E-05	2.59583E-05	1.47274E-05	1.58545E-05	0.02019729	0.36000697
22	2',6'-Dihydroxy 4'-methoxydihydrochalcone	+	10.72	1.36668E-05	2.19204E-05	8.61159E-06	1.34221E-05	0.02103828	0.36000697
23	Gallic acid	-	6.07	4.25894E-05	4.05937E-05	2.19085E-05	3.71679E-05	0.021928976	0.36000697
24	Cauloside A	+	7.4	4.40184E-06	1.14506E-06	4.86118E-06	1.45974E-06	0.022233734	0.36000697
25	Irisflorentin	+	8.64	9.3776E-05	7.82073E-05	4.62322E-05	3.28347E-05	0.023308723	0.36000697
26	1,3,6-Tri-O-galloylglucose	+	5.79	0.000378633	0.000384123	0.000216121	0.000239668	0.023400453	0.36000697
27	Physcion	+	8.24	1.31602E-05	6.56822E-06	4.70428E-06	5.64191E-06	0.02515224	0.372625773
28	1-(4-Hydroxyphenyl)propan-1-one	+	9.9	0.000102169	4.64683E-05	5.61041E-05	4.35277E-05	0.027091226	0.387017517
29	2'-Hydroxygenistein	+	7.59	1.20806E-05	1.67422E-05	3.28747E-05	1.96421E-05	0.033523805	0.462397304
30	4-Isopropylbenzoic acid	+	9	0.000108511	8.14101E-05	6.40269E-05	6.01538E-05	0.036432729	0.468058572
31	4-Methoxycinnamaldehyde	+	6.8	3.71837E-05	4.28742E-05	2.29892E-05	2.49645E-05	0.036717917	0.468058572
32	L-Phenylalanine;D-(+)-Phenylalanin	+	2.82	0.034211697	0.045900681	0.049091859	0.048253966	0.0385504	0.468058572
33	Epicatechin;(+)-Epicatechin	+	5.08	0.000213322	0.000305891	0.000180495	8.02881E-05	0.038614832	0.468058572
34	N-D-Glucosylarylamine	+	2.69	0.00022197	0.000104612	0.000150671	0.000118153	0.041023159	0.482625403
35	p-Cresol	+	7.28	4.39354E-05	3.8862E-05	3.51015E-05	3.36452E-05	0.04475265	0.511458863
36	Abyssinone V	+	12.6	2.83944E-05	2.6629E-05	3.09344E-05	3.59097E-05	0.049678281	0.52815576
37	Tropine acetate;3-Acetoxytropane	+	3.24	6.02128E-05	8.11415E-05	5.12088E-05	6.92808E-05	0.049760477	0.52815576

Note: Ion: Positive and negative ion modes of mass spectrometry; Rt: Chromatographic retention time of this substance; Mean CK, Mean S, Mean S40, and Mean S40+S: The relative quantitative mean value of different groups; *P*-VALUE: *P* value of the variance of the substance in the group comparison; Q-VALUE: The result of hypothesis test statistics (*P* value) corrected by multiple hypothesis tests.
